# Supplementary material for: Treating Withdrawal and Pain in Inpatients With Opioid Use Disorder: A Brief Educational Intervention for Internal Medicine Residents
Source: MedEdPORTAL. 2021 Mar 10;17:11123. doi: 10.15766/mep_2374-8265.11123 (PMC7970646; doi:10.15766/mep_2374-8265.11123)
Supplement: Supplementary file 1 — Presentation Materials.pptxPre- and Postsurvey.docx [file mep_2374-8265.11123-s001.zip › A. Presentation Materials.pptx]

## Slide 1
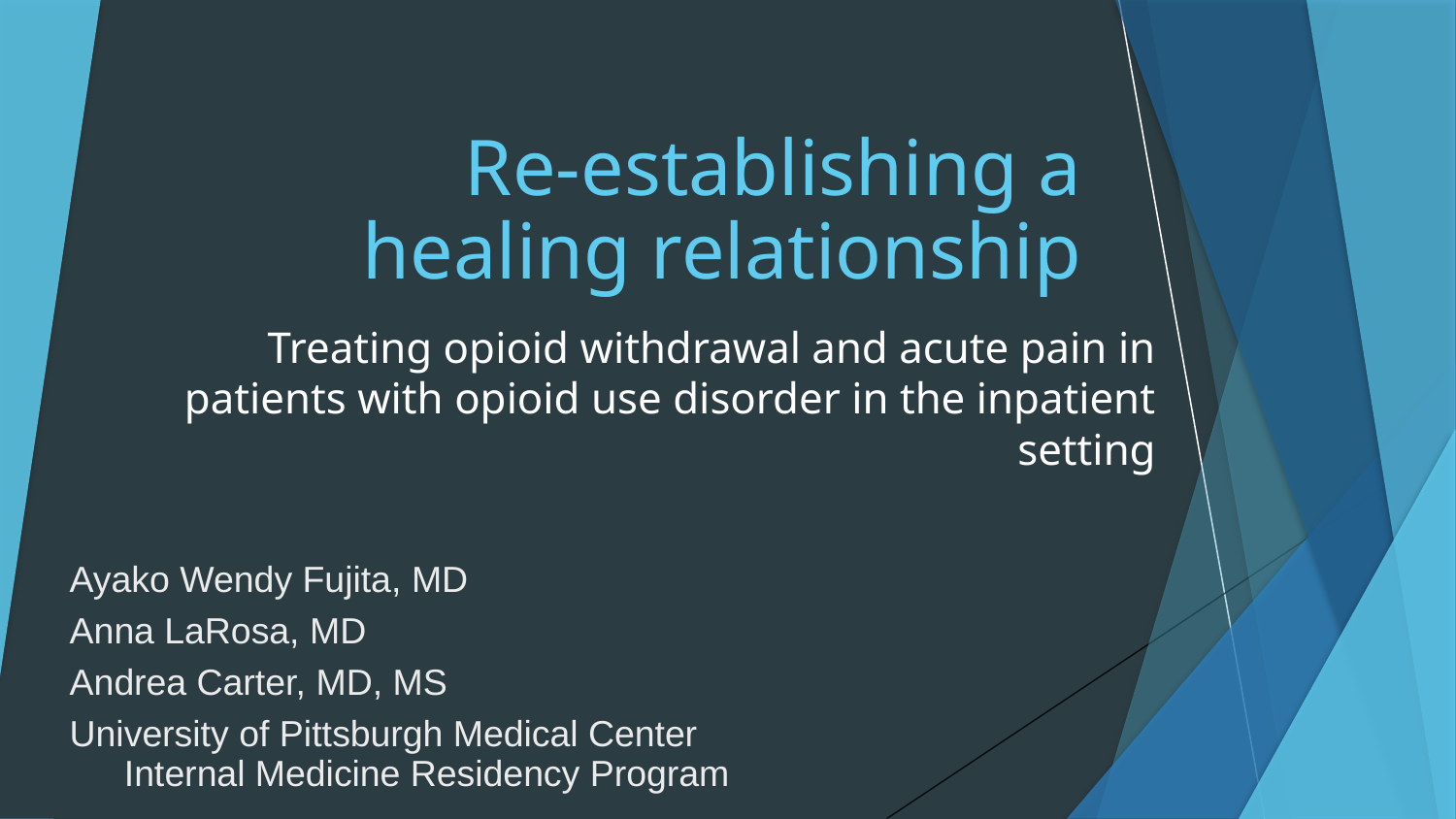

# Re-establishing a healing relationship
Treating opioid withdrawal and acute pain in patients with opioid use disorder in the inpatient setting
Ayako Wendy Fujita, MD
Anna LaRosa, MD
Andrea Carter, MD, MS
University of Pittsburgh Medical Center Internal Medicine Residency Program

## Slide 2
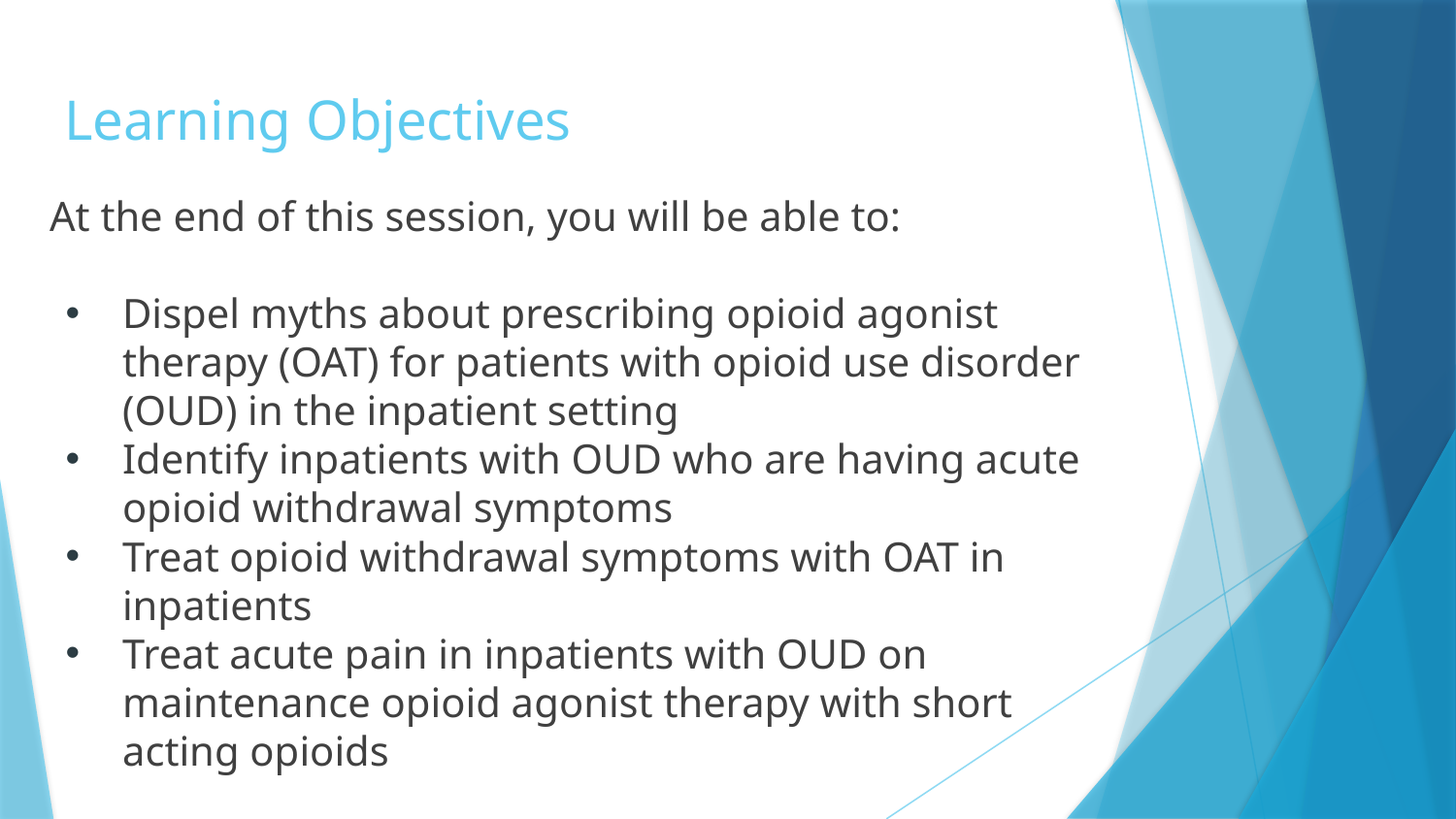

# Learning Objectives
At the end of this session, you will be able to:
Dispel myths about prescribing opioid agonist therapy (OAT) for patients with opioid use disorder (OUD) in the inpatient setting
Identify inpatients with OUD who are having acute opioid withdrawal symptoms
Treat opioid withdrawal symptoms with OAT in inpatients
Treat acute pain in inpatients with OUD on maintenance opioid agonist therapy with short acting opioids

## Slide 3
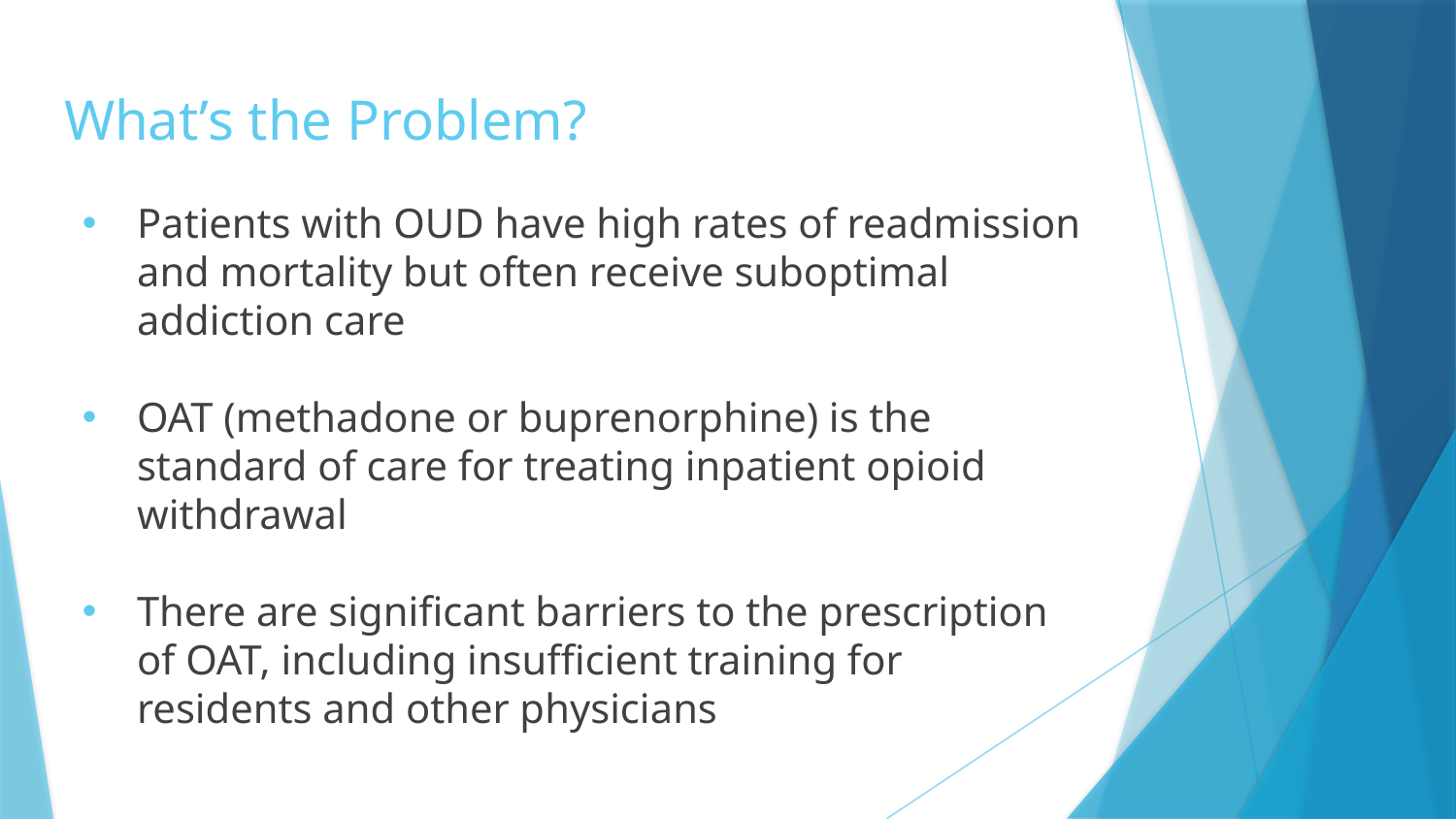

# What’s the Problem?
Patients with OUD have high rates of readmission and mortality but often receive suboptimal addiction care
OAT (methadone or buprenorphine) is the standard of care for treating inpatient opioid withdrawal
There are significant barriers to the prescription of OAT, including insufficient training for residents and other physicians

## Slide 4
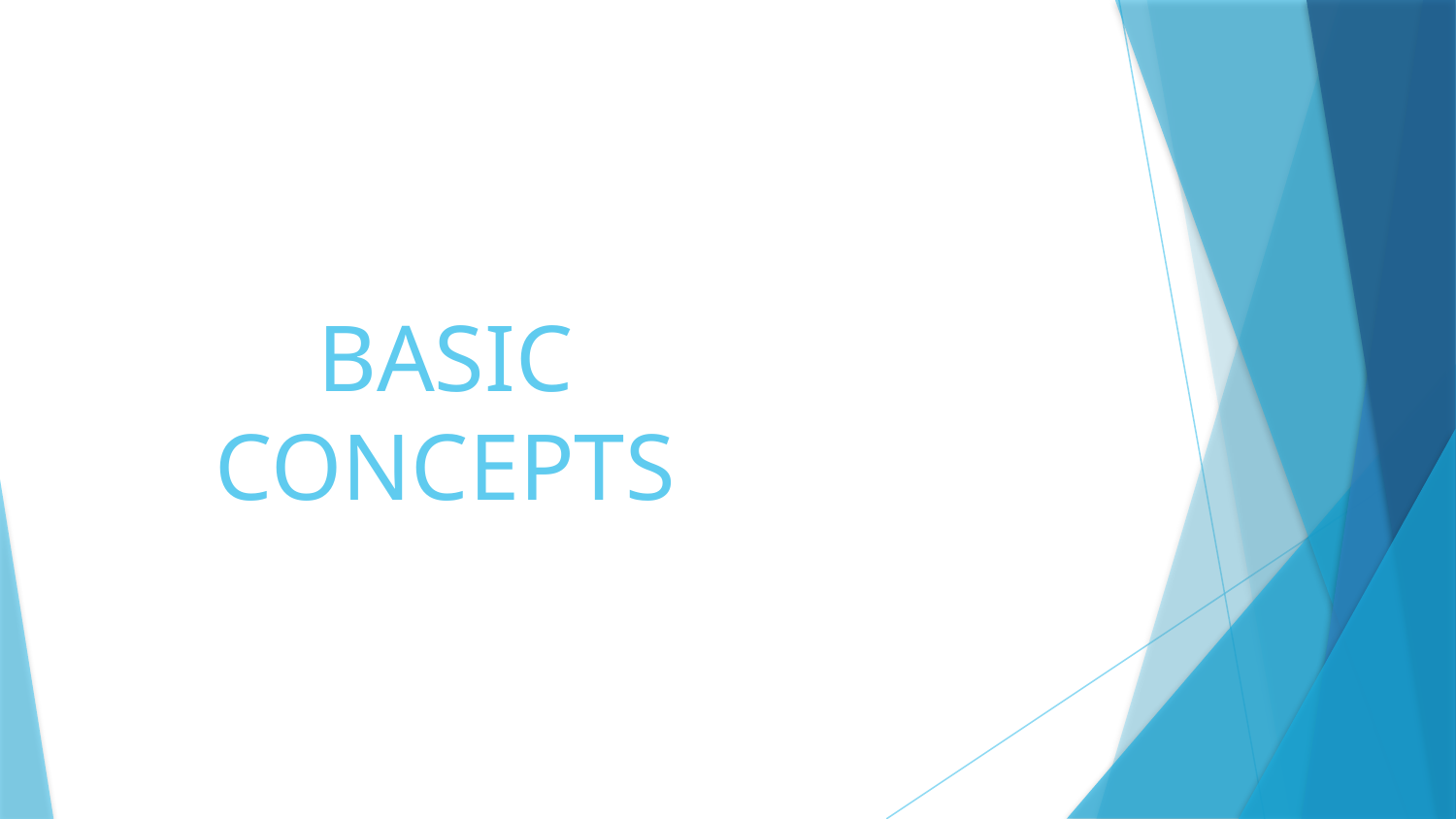

# BASIC CONCEPTS

## Slide 5
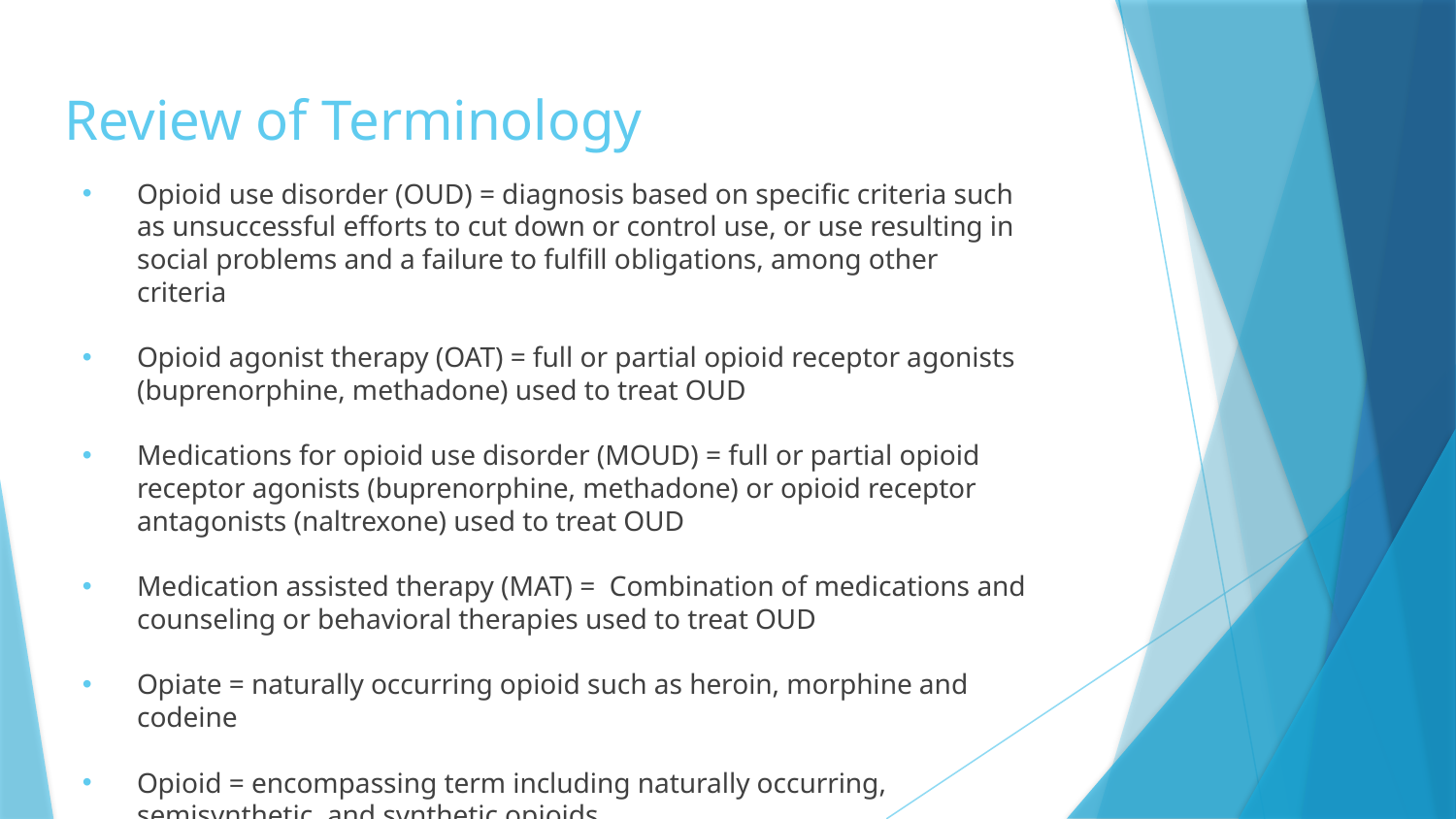

# Review of Terminology
Opioid use disorder (OUD) = diagnosis based on specific criteria such as unsuccessful efforts to cut down or control use, or use resulting in social problems and a failure to fulfill obligations, among other criteria
Opioid agonist therapy (OAT) = full or partial opioid receptor agonists (buprenorphine, methadone) used to treat OUD
Medications for opioid use disorder (MOUD) = full or partial opioid receptor agonists (buprenorphine, methadone) or opioid receptor antagonists (naltrexone) used to treat OUD
Medication assisted therapy (MAT) = Combination of medications and counseling or behavioral therapies used to treat OUD
Opiate = naturally occurring opioid such as heroin, morphine and codeine
Opioid = encompassing term including naturally occurring, semisynthetic, and synthetic opioids

## Slide 6
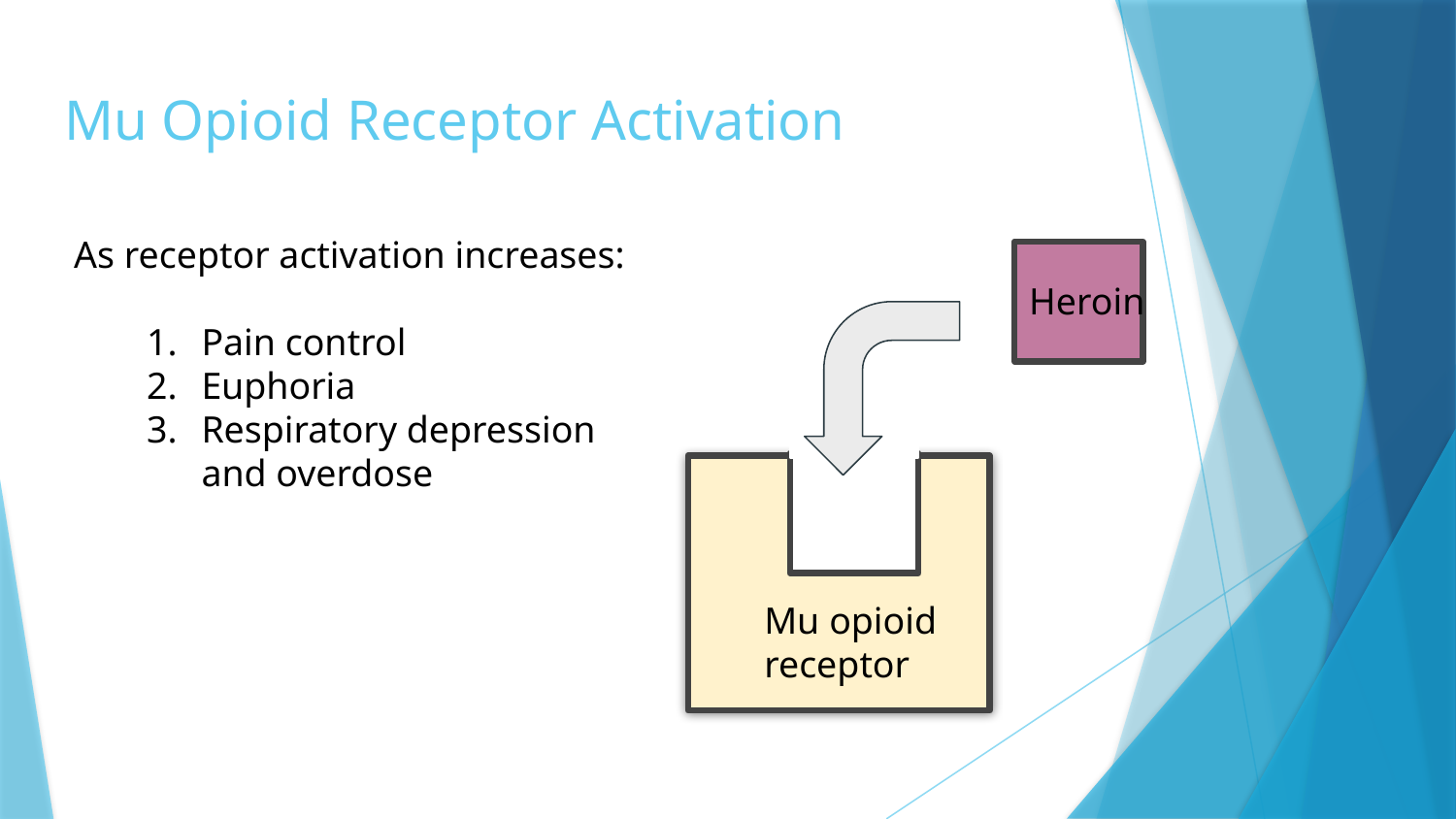

# Mu Opioid Receptor Activation
As receptor activation increases:
Pain control
Euphoria
Respiratory depression and overdose
Heroin
Mu opioid receptor

## Slide 7
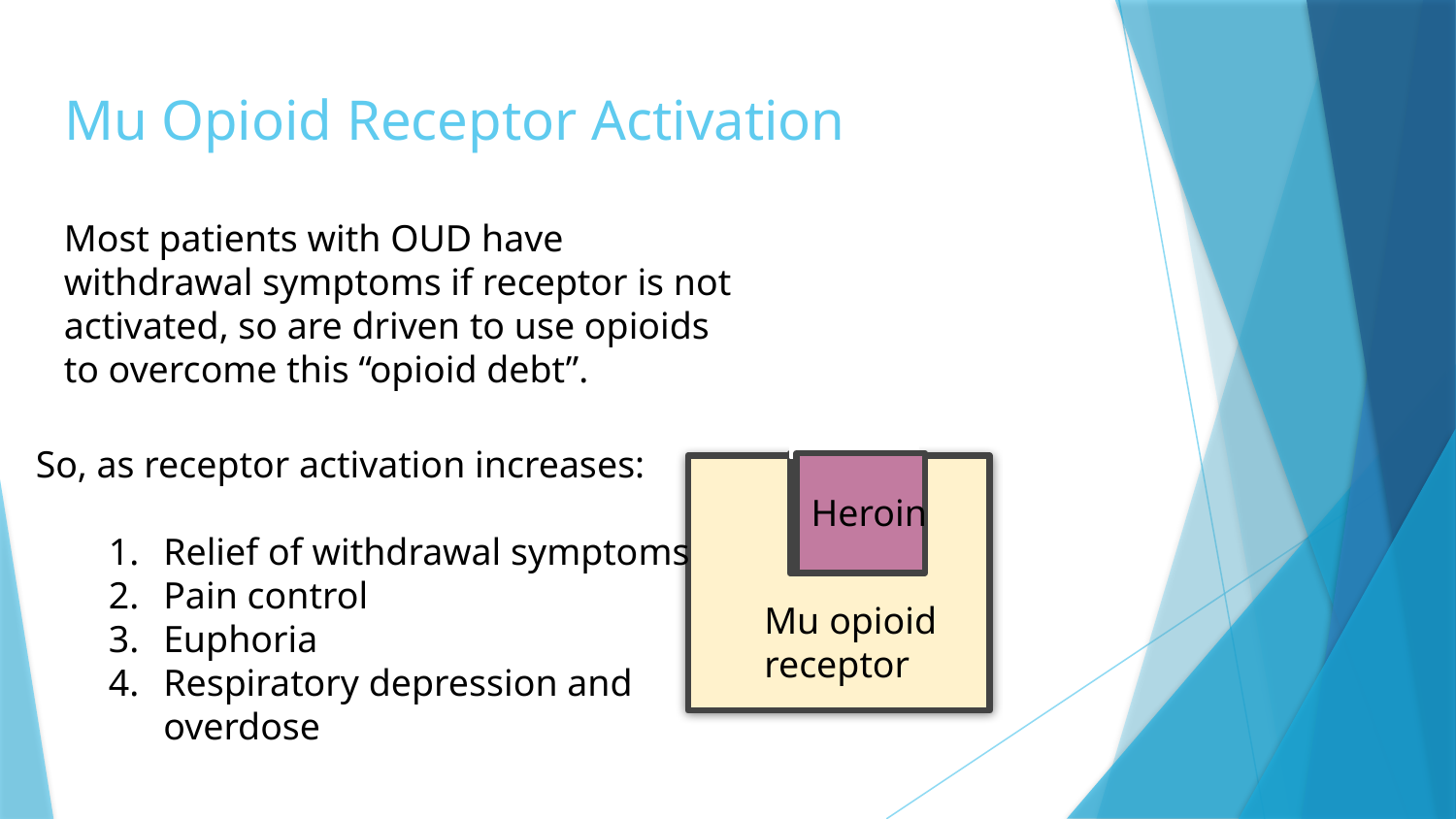

# Mu Opioid Receptor Activation
Most patients with OUD have withdrawal symptoms if receptor is not activated, so are driven to use opioids to overcome this “opioid debt”.
So, as receptor activation increases:
Relief of withdrawal symptoms
Pain control
Euphoria
Respiratory depression and overdose
Heroin
Mu opioid receptor

## Slide 8
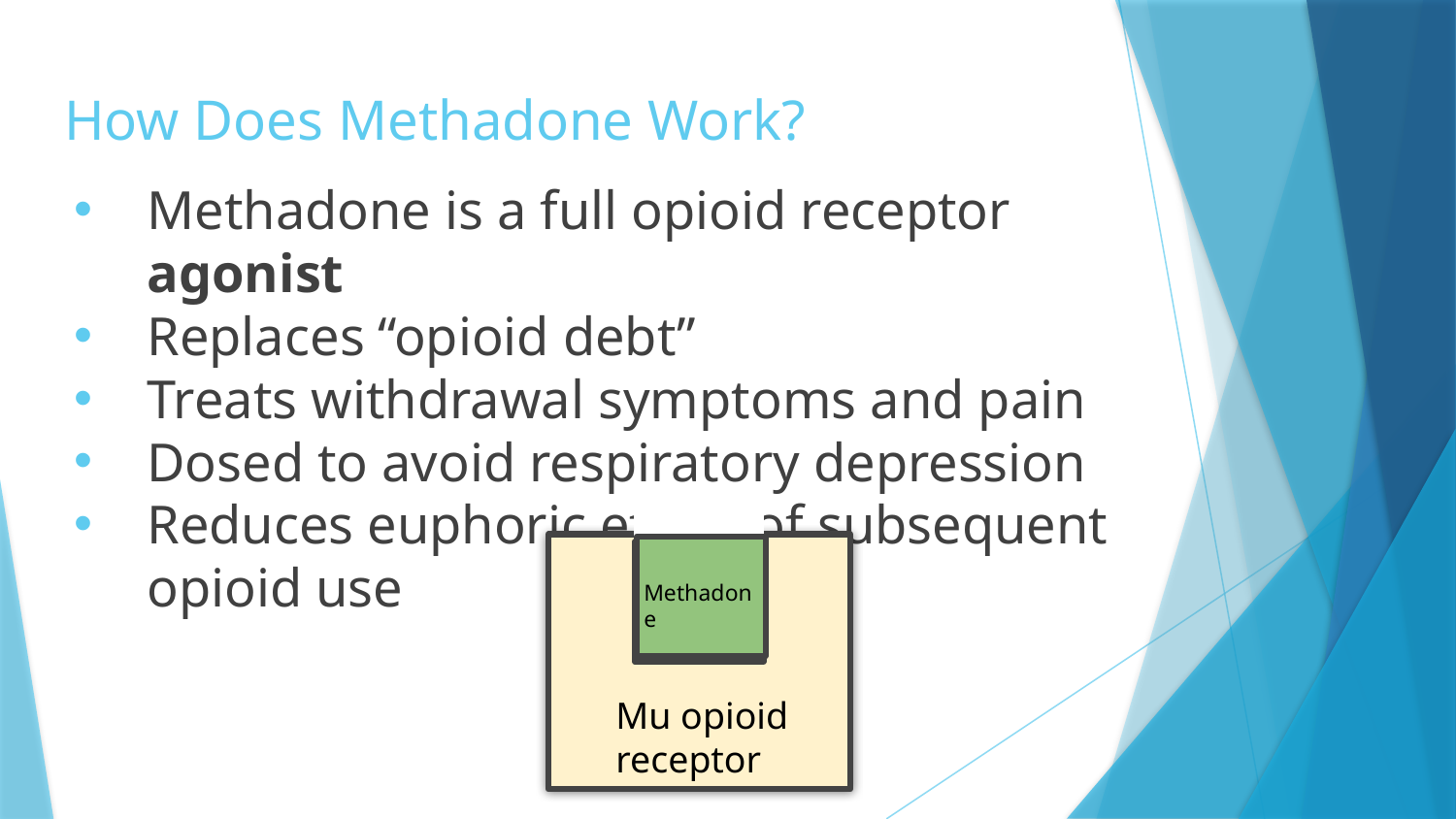

# How Does Methadone Work?
Methadone is a full opioid receptor agonist
Replaces “opioid debt”
Treats withdrawal symptoms and pain
Dosed to avoid respiratory depression
Reduces euphoric effect of subsequent opioid use
Methadone
Mu opioid receptor

## Slide 9
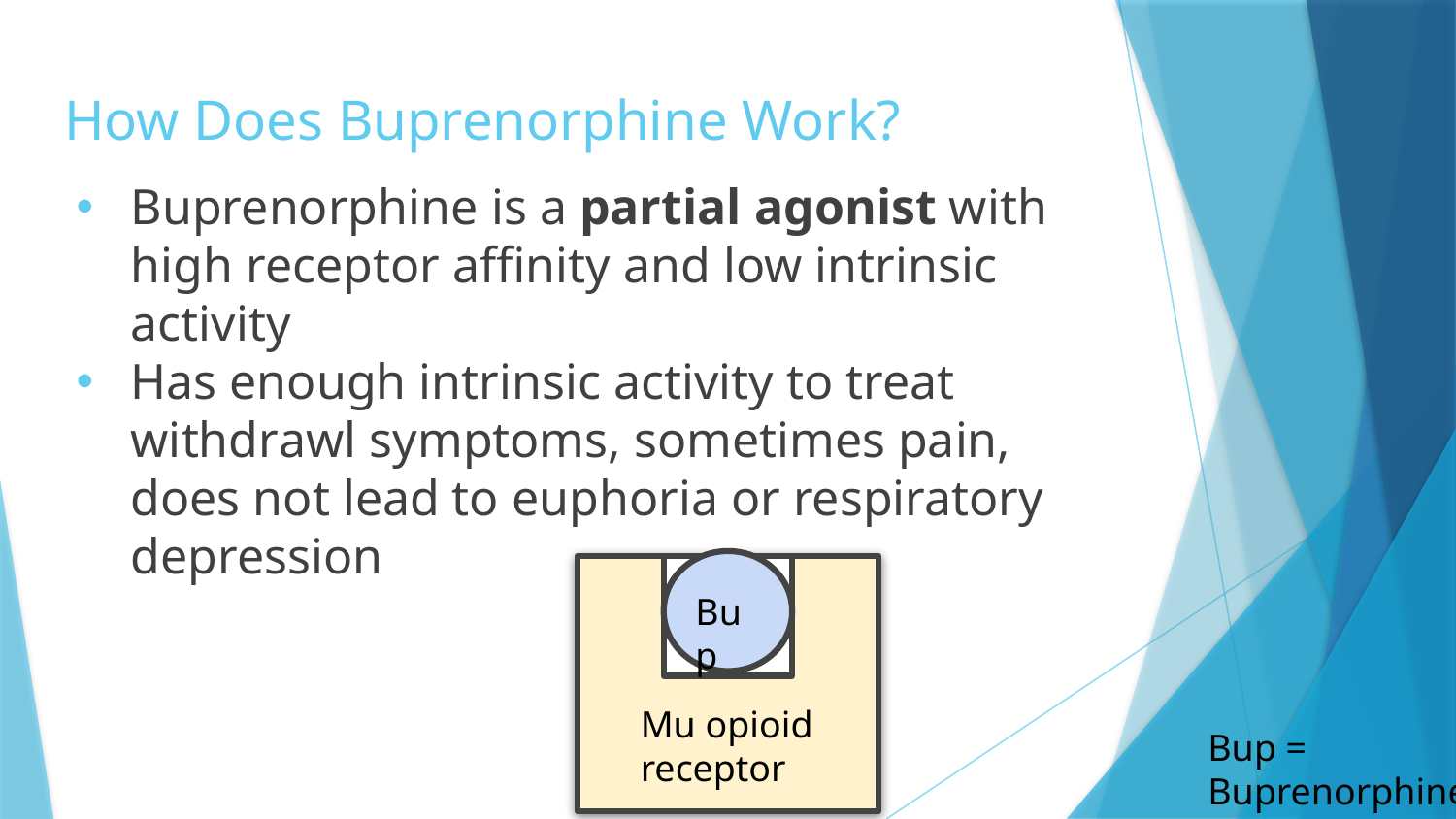

# How Does Buprenorphine Work?
Buprenorphine is a partial agonist with high receptor affinity and low intrinsic activity
Has enough intrinsic activity to treat withdrawl symptoms, sometimes pain, does not lead to euphoria or respiratory depression
Bup
Mu opioid receptor
Bup = Buprenorphine

## Slide 10
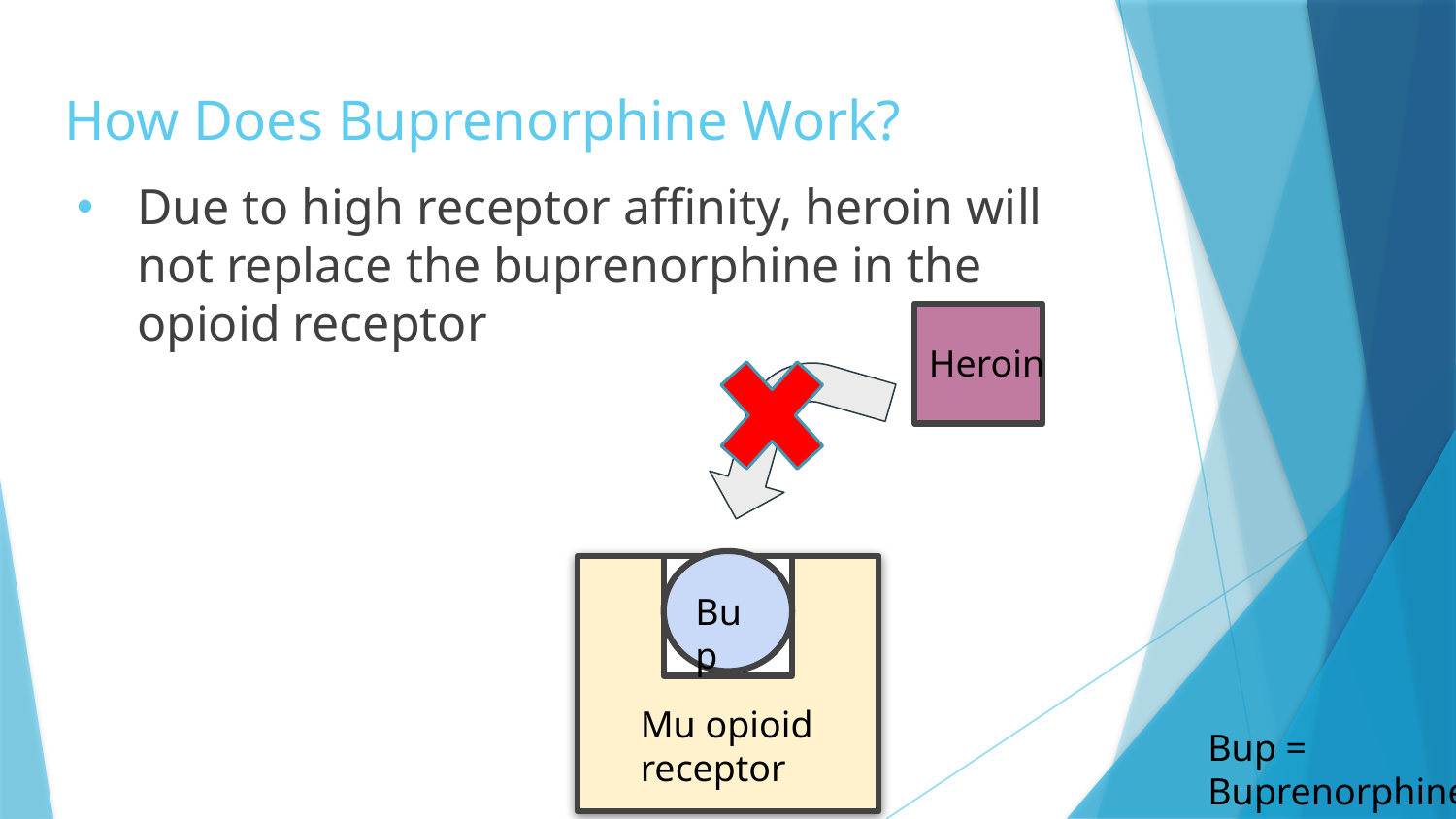

# How Does Buprenorphine Work?
Due to high receptor affinity, heroin will not replace the buprenorphine in the opioid receptor
Heroin
Bup
Mu opioid receptor
Bup = Buprenorphine

## Slide 11
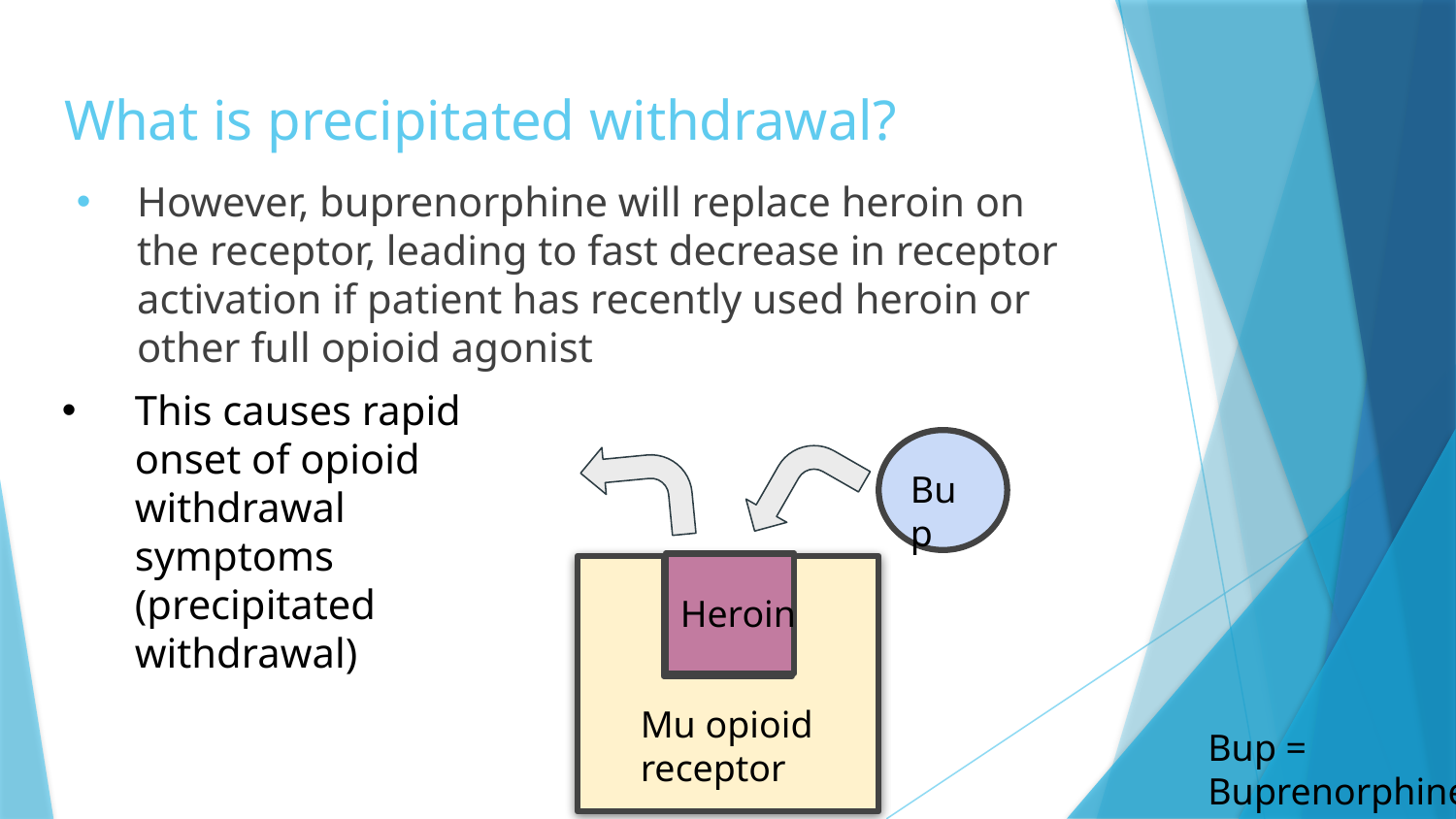

# What is precipitated withdrawal?
However, buprenorphine will replace heroin on the receptor, leading to fast decrease in receptor activation if patient has recently used heroin or other full opioid agonist
This causes rapid onset of opioid withdrawal symptoms (precipitated withdrawal)
Bup
Heroin
Mu opioid receptor
Bup = Buprenorphine

## Slide 12
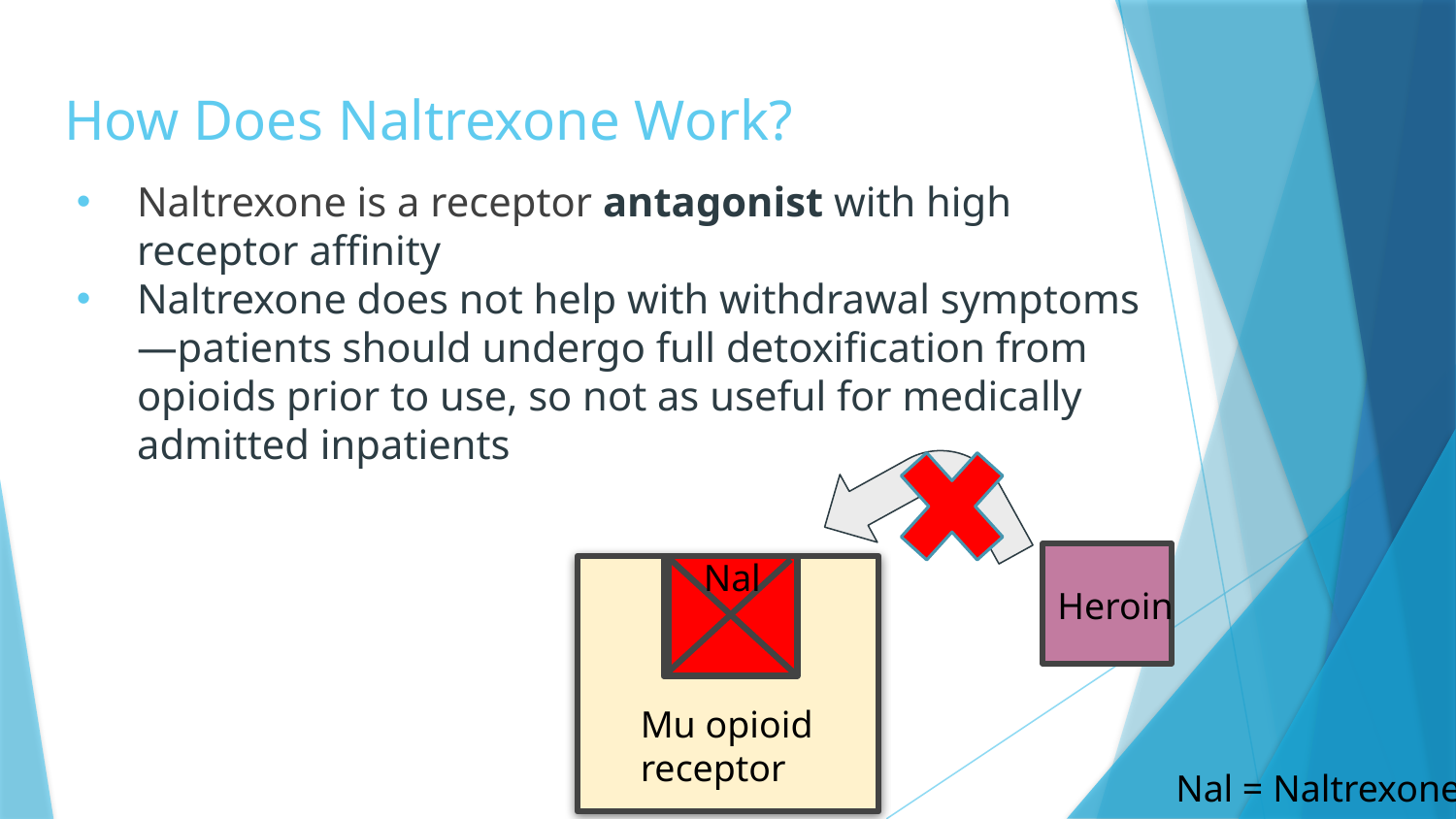

# How Does Naltrexone Work?
Naltrexone is a receptor antagonist with high receptor affinity
Naltrexone does not help with withdrawal symptoms—patients should undergo full detoxification from opioids prior to use, so not as useful for medically admitted inpatients
Nal
Heroin
Mu opioid receptor
Nal = Naltrexone

## Slide 13
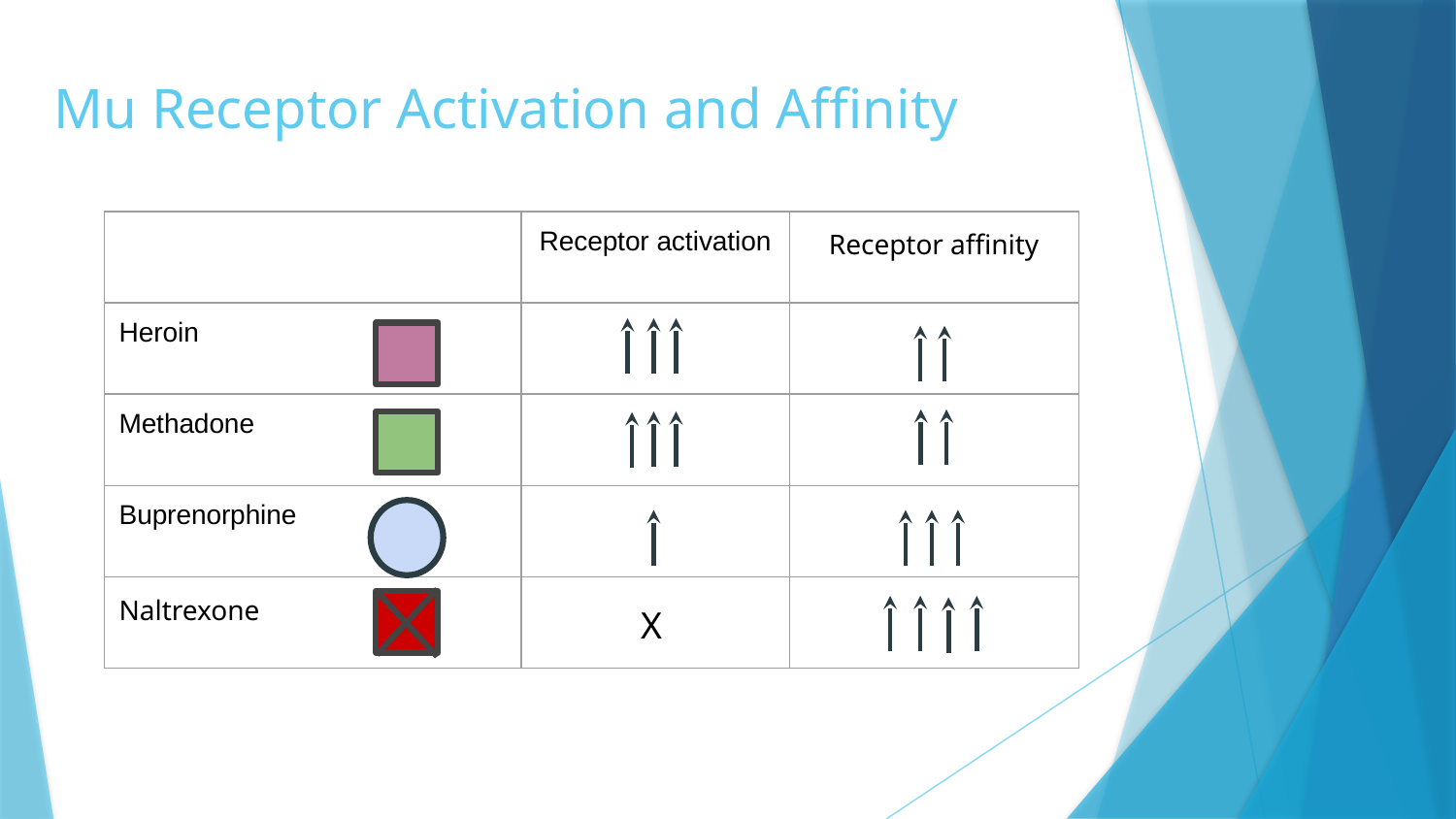

# Mu Receptor Activation and Affinity
| | Receptor activation | Receptor affinity |
| --- | --- | --- |
| Heroin | | |
| Methadone | | |
| Buprenorphine | | |
| Naltrexone | | |
X

## Slide 14
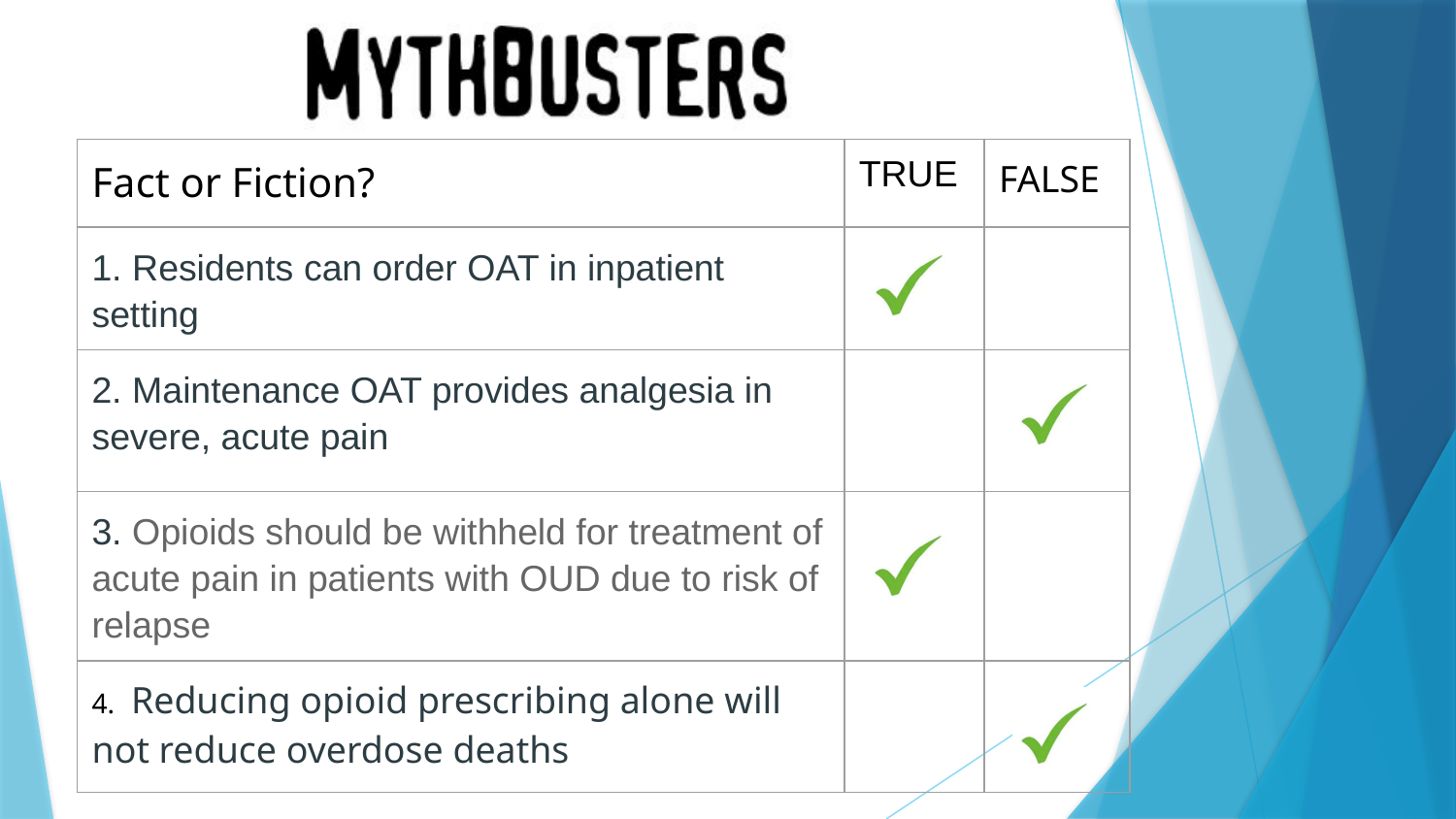

| Fact or Fiction? | TRUE | FALSE |
| --- | --- | --- |
| 1. Residents can order OAT in inpatient setting | | |
| 2. Maintenance OAT provides analgesia in severe, acute pain | | |
| 3. Opioids should be withheld for treatment of acute pain in patients with OUD due to risk of relapse | | |
| 4. Reducing opioid prescribing alone will not reduce overdose deaths | | |

## Slide 15
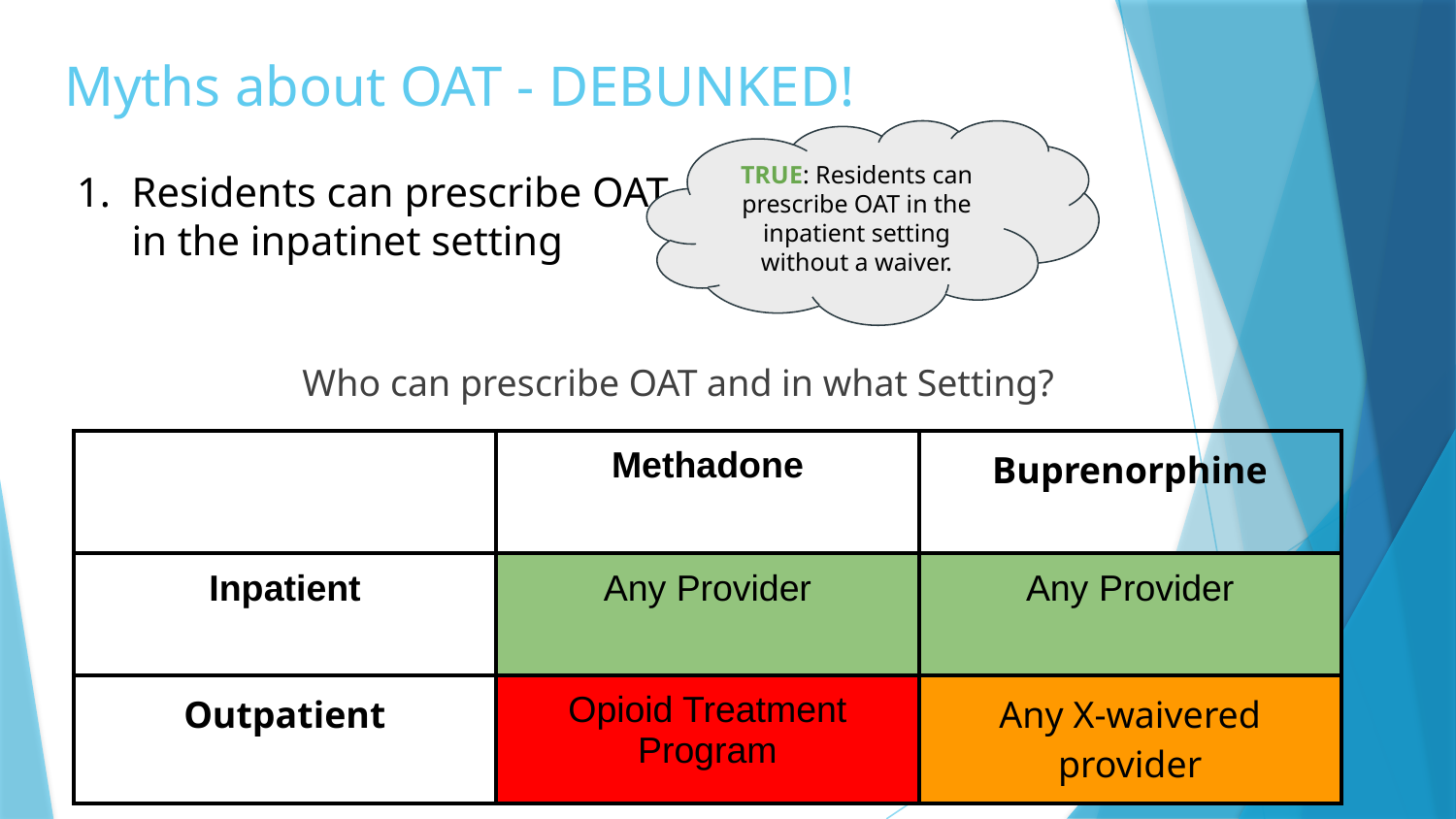

# Myths about OAT - DEBUNKED!
TRUE: Residents can prescribe OAT in the inpatient setting without a waiver.
Residents can prescribe OAT in the inpatinet setting
Who can prescribe OAT and in what Setting?
| | Methadone | Buprenorphine |
| --- | --- | --- |
| Inpatient | Any Provider | Any Provider |
| Outpatient | Opioid Treatment Program | Any X-waivered provider |

## Slide 16
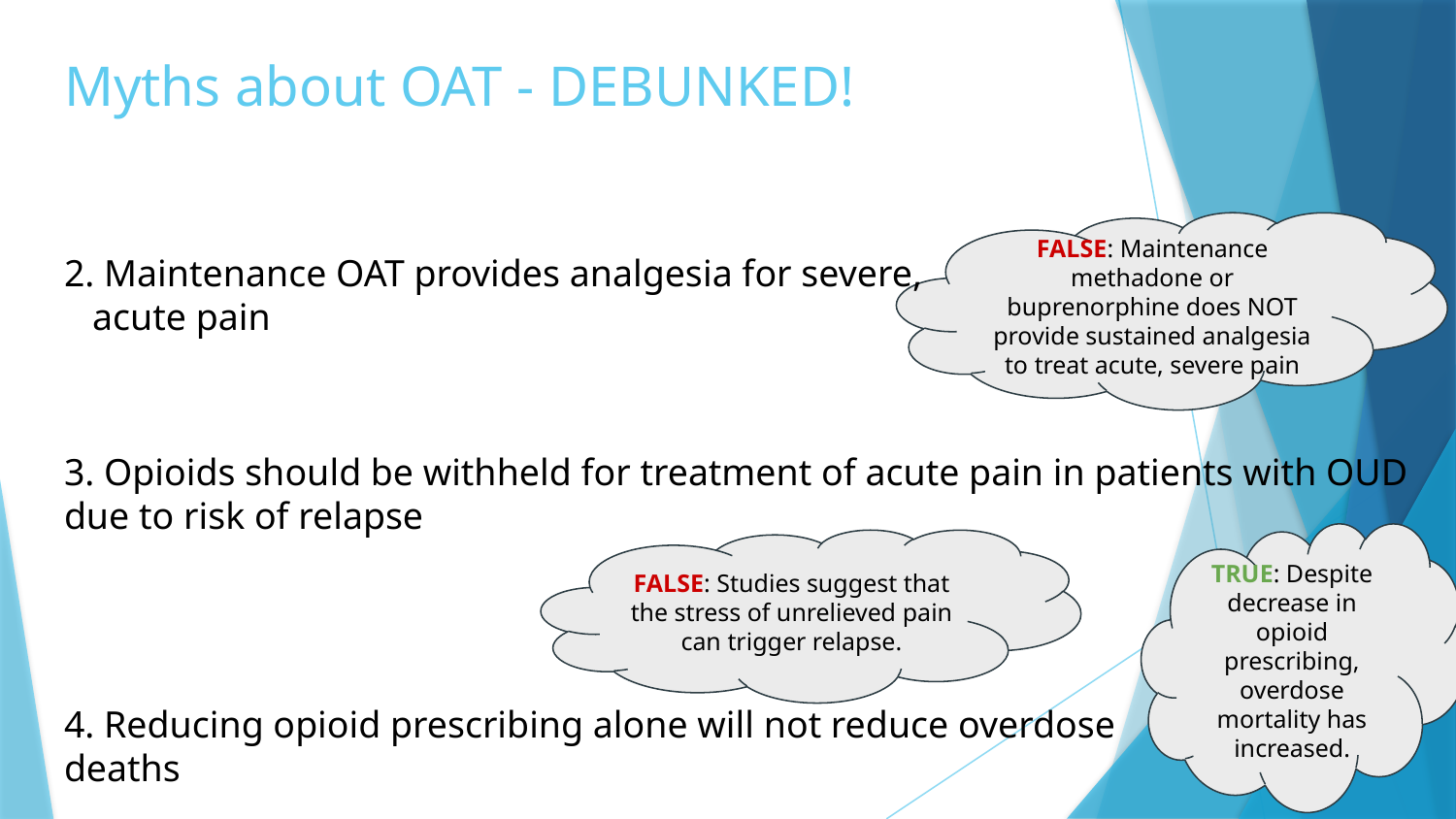

# Myths about OAT - DEBUNKED!
FALSE: Maintenance methadone or buprenorphine does NOT provide sustained analgesia to treat acute, severe pain
2. Maintenance OAT provides analgesia for severe, acute pain
3. Opioids should be withheld for treatment of acute pain in patients with OUD due to risk of relapse
TRUE: Despite decrease in opioid prescribing, overdose mortality has increased.
FALSE: Studies suggest that the stress of unrelieved pain can trigger relapse.
4. Reducing opioid prescribing alone will not reduce overdose deaths

## Slide 17
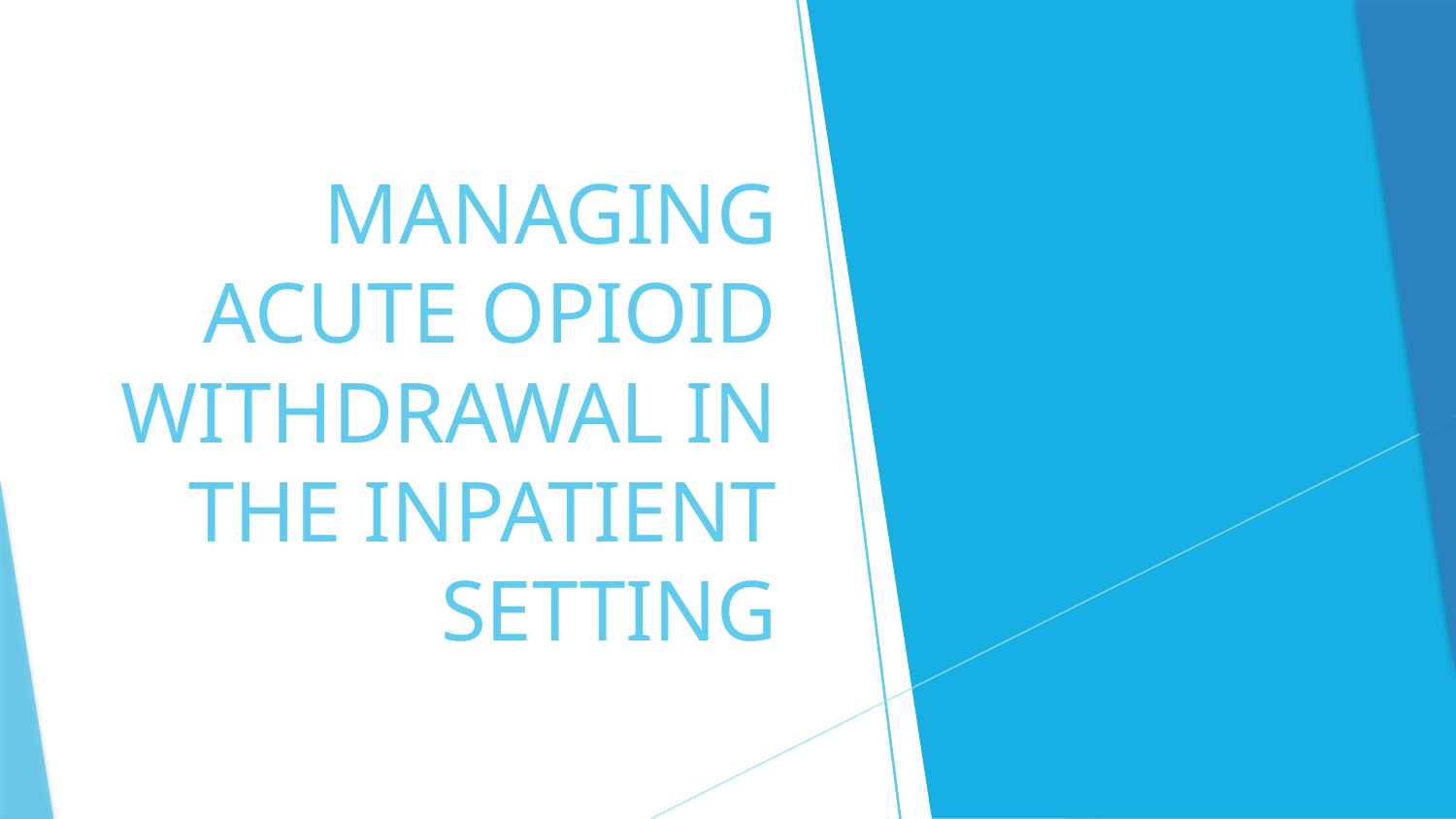

# MANAGING ACUTE OPIOID WITHDRAWAL IN THE INPATIENT SETTING

## Slide 18
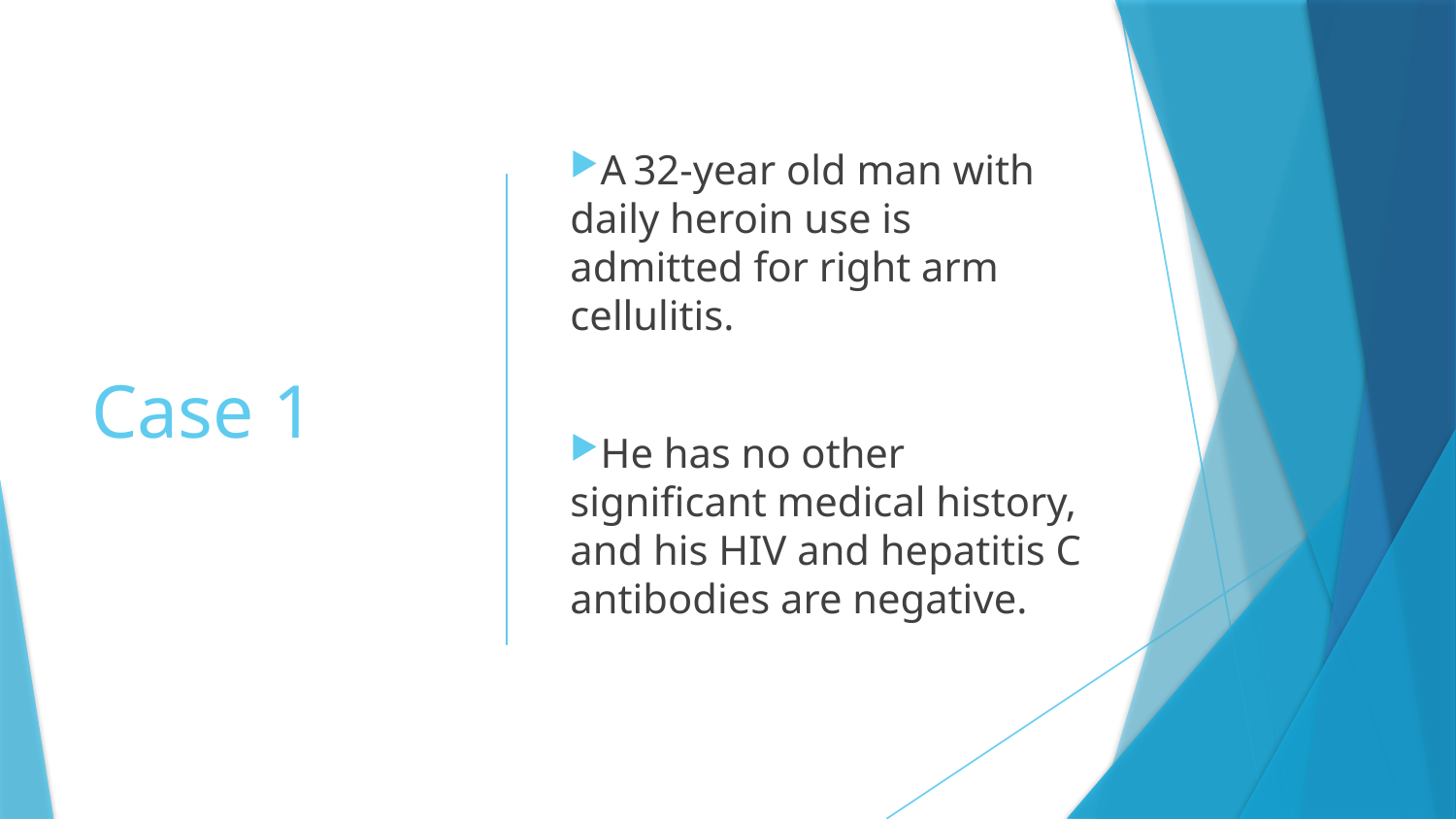

# Case 1
A 32-year old man with daily heroin use is admitted for right arm cellulitis.
He has no other significant medical history, and his HIV and hepatitis C antibodies are negative.

## Slide 19
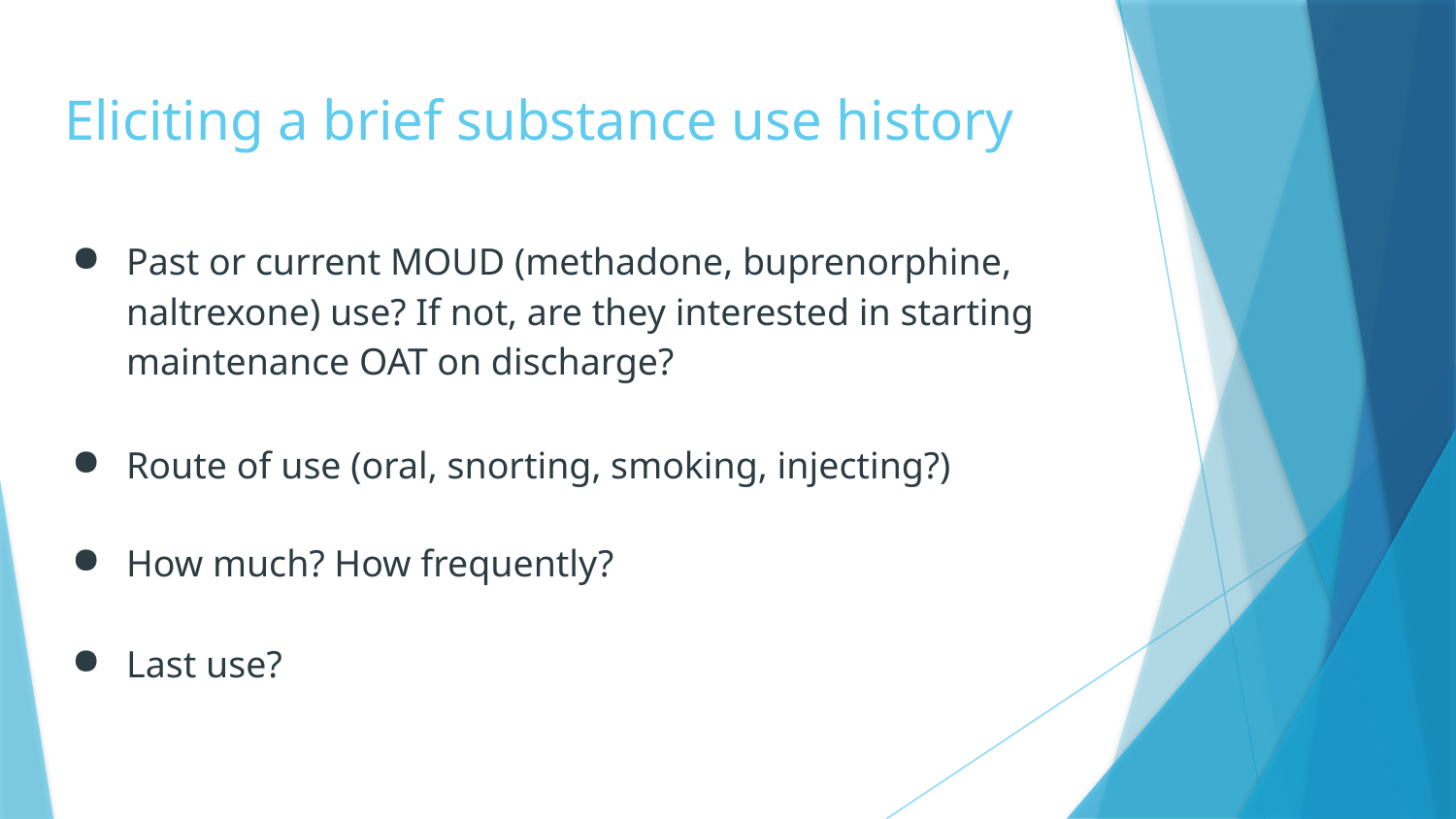

# Eliciting a brief substance use history
Past or current MOUD (methadone, buprenorphine, naltrexone) use? If not, are they interested in starting maintenance OAT on discharge?
Route of use (oral, snorting, smoking, injecting?)
How much? How frequently?
Last use?

## Slide 20
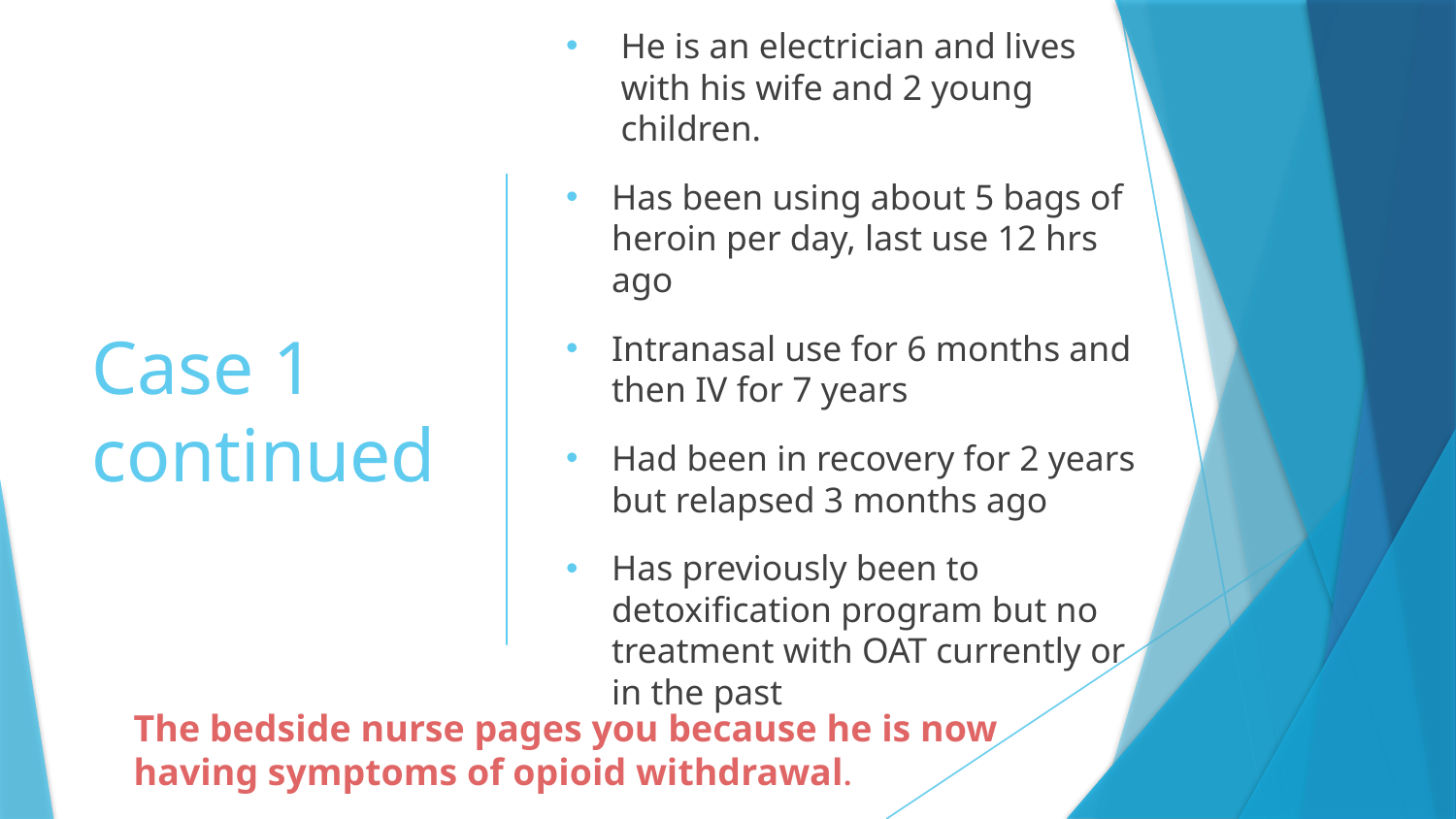

He is an electrician and lives with his wife and 2 young children.
Has been using about 5 bags of heroin per day, last use 12 hrs ago
Intranasal use for 6 months and then IV for 7 years
Had been in recovery for 2 years but relapsed 3 months ago
Has previously been to detoxification program but no treatment with OAT currently or in the past
# Case 1 continued
The bedside nurse pages you because he is now having symptoms of opioid withdrawal.

## Slide 21
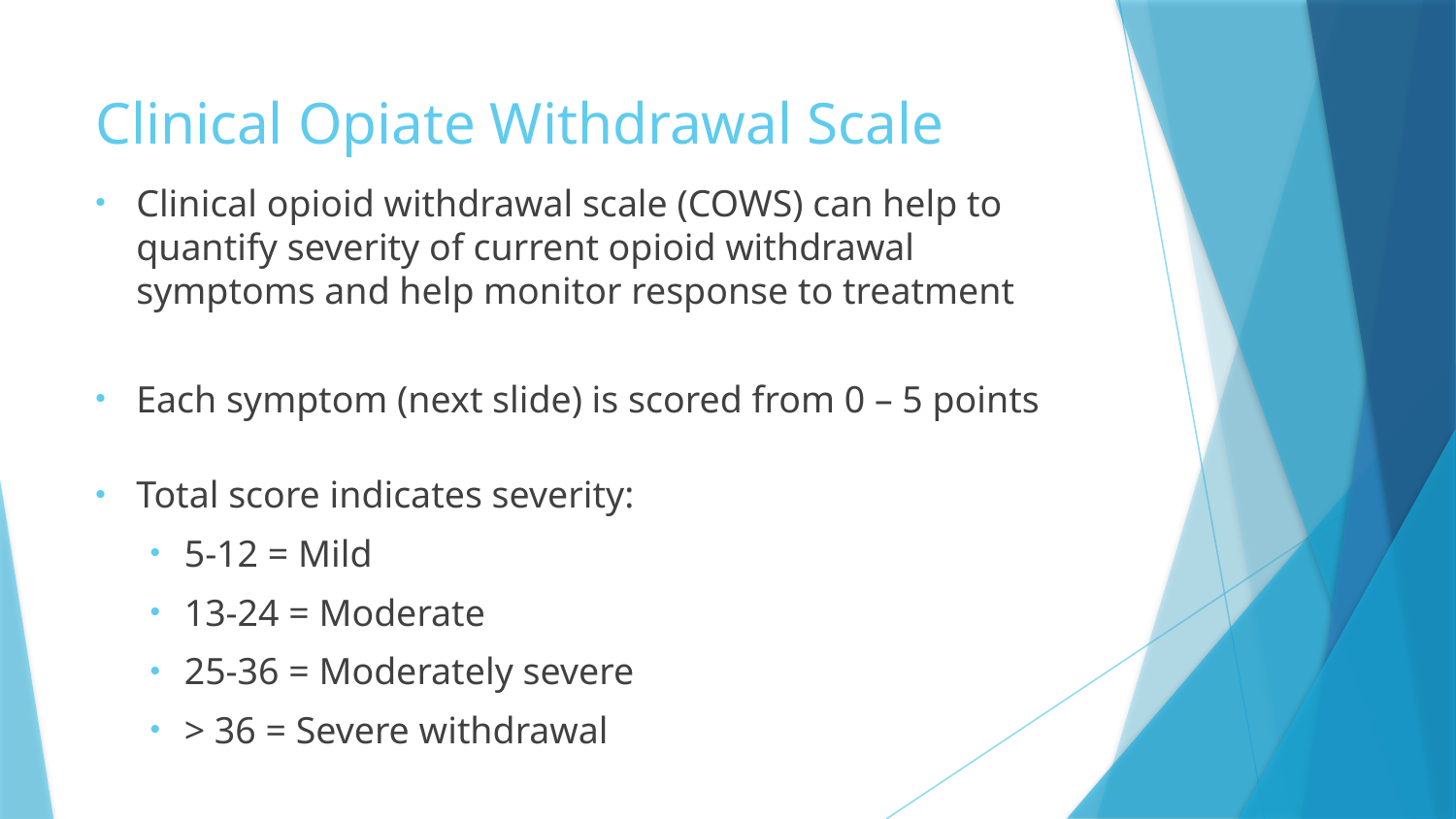

# Clinical Opiate Withdrawal Scale
Clinical opioid withdrawal scale (COWS) can help to quantify severity of current opioid withdrawal symptoms and help monitor response to treatment
Each symptom (next slide) is scored from 0 – 5 points
Total score indicates severity:
5-12 = Mild
13-24 = Moderate
25-36 = Moderately severe
> 36 = Severe withdrawal

## Slide 22
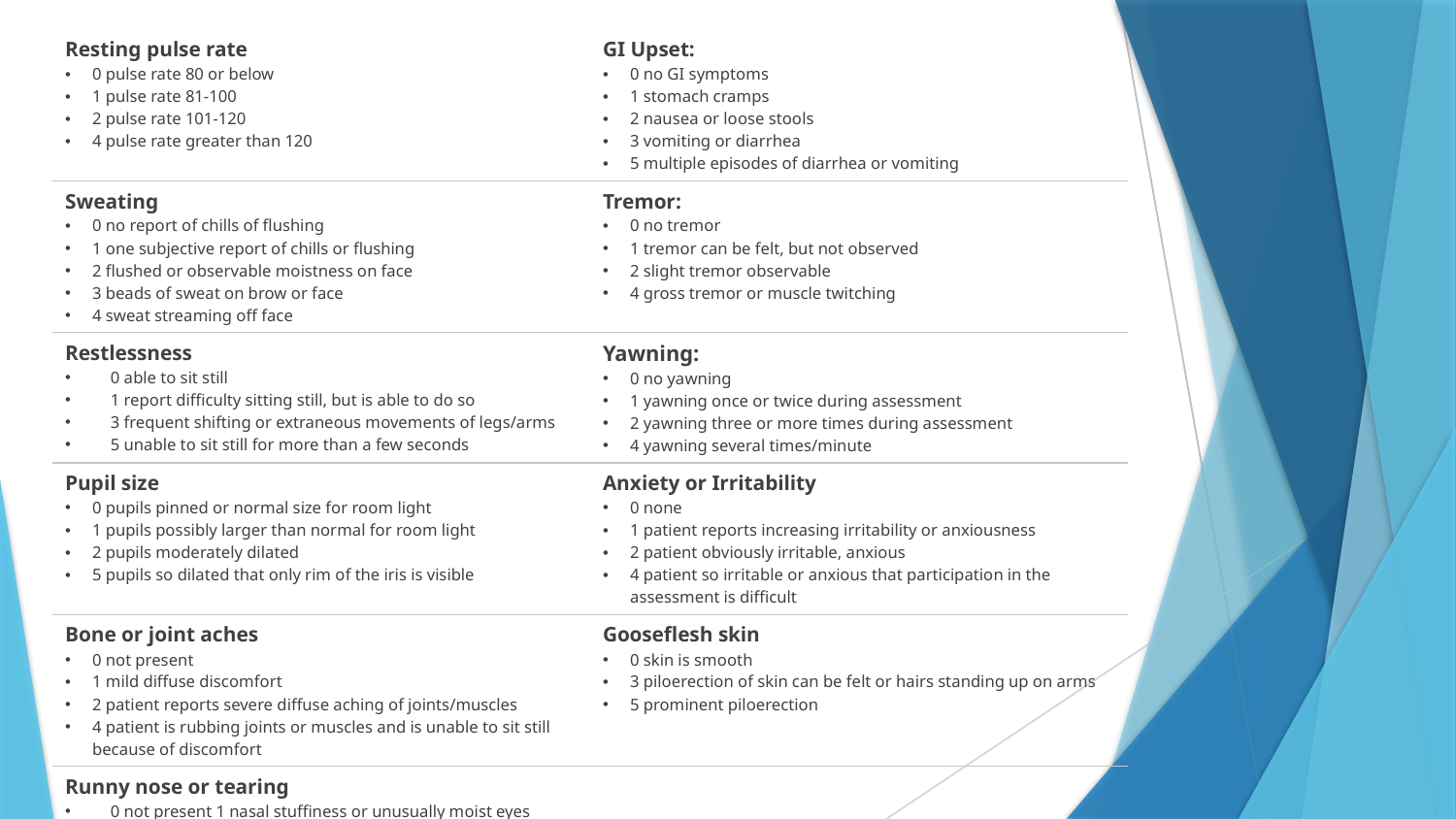

| Resting pulse rate 0 pulse rate 80 or below 1 pulse rate 81-100 2 pulse rate 101-120 4 pulse rate greater than 120 | GI Upset: 0 no GI symptoms 1 stomach cramps 2 nausea or loose stools 3 vomiting or diarrhea 5 multiple episodes of diarrhea or vomiting |
| --- | --- |
| Sweating 0 no report of chills of flushing 1 one subjective report of chills or flushing 2 flushed or observable moistness on face 3 beads of sweat on brow or face 4 sweat streaming off face | Tremor: 0 no tremor 1 tremor can be felt, but not observed 2 slight tremor observable 4 gross tremor or muscle twitching |
| Restlessness 0 able to sit still 1 report difficulty sitting still, but is able to do so 3 frequent shifting or extraneous movements of legs/arms 5 unable to sit still for more than a few seconds | Yawning: 0 no yawning 1 yawning once or twice during assessment 2 yawning three or more times during assessment 4 yawning several times/minute |
| Pupil size 0 pupils pinned or normal size for room light 1 pupils possibly larger than normal for room light 2 pupils moderately dilated 5 pupils so dilated that only rim of the iris is visible | Anxiety or Irritability 0 none 1 patient reports increasing irritability or anxiousness 2 patient obviously irritable, anxious 4 patient so irritable or anxious that participation in the assessment is difficult |
| Bone or joint aches 0 not present 1 mild diffuse discomfort 2 patient reports severe diffuse aching of joints/muscles 4 patient is rubbing joints or muscles and is unable to sit still because of discomfort | Gooseflesh skin 0 skin is smooth 3 piloerection of skin can be felt or hairs standing up on arms 5 prominent piloerection |
| Runny nose or tearing 0 not present 1 nasal stuffiness or unusually moist eyes 2 nose running or tearing 4 nose constantly running or tears streaming down cheeks | |

## Slide 23
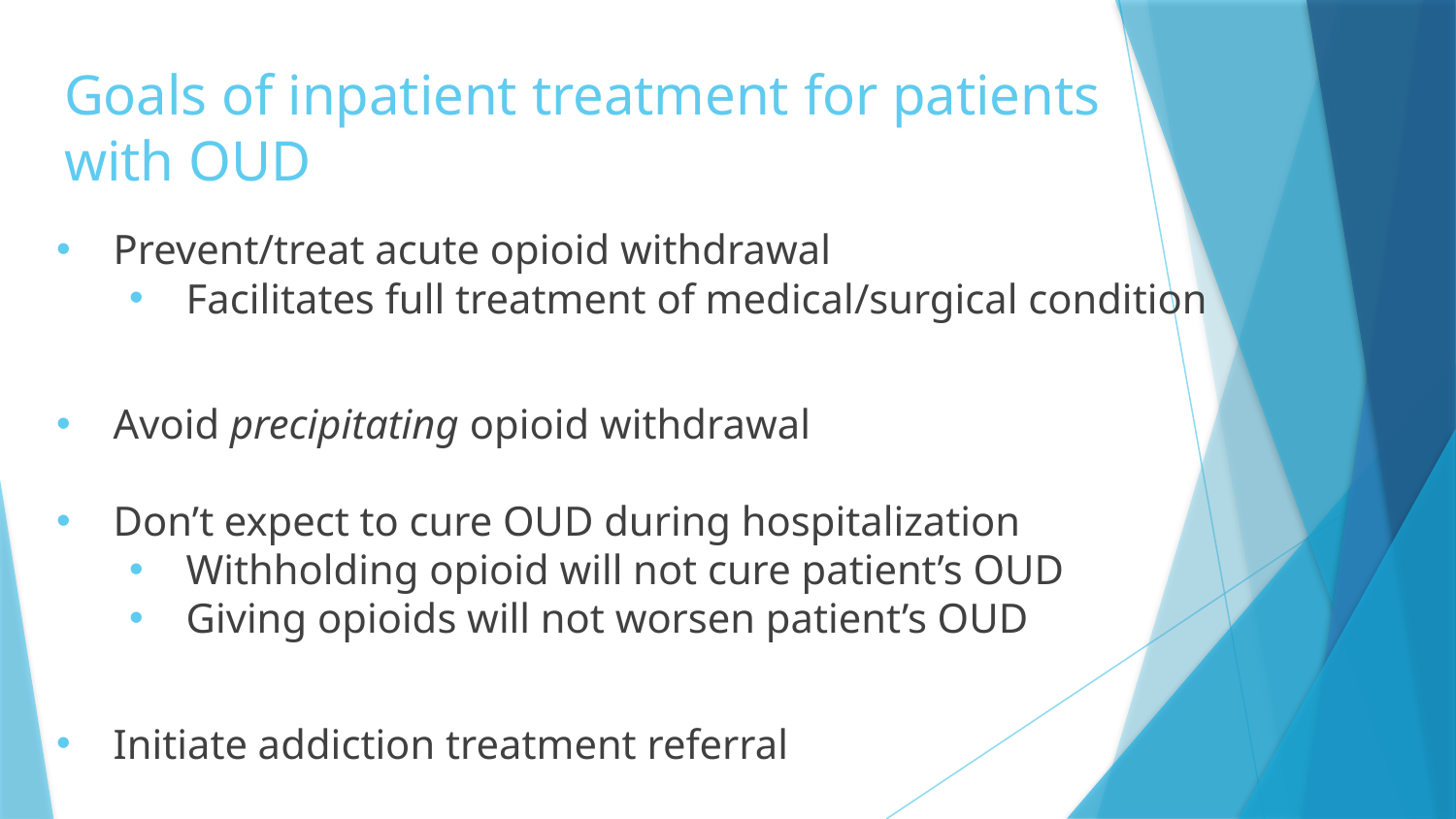

# Goals of inpatient treatment for patients with OUD
Prevent/treat acute opioid withdrawal
Facilitates full treatment of medical/surgical condition
Avoid precipitating opioid withdrawal
Don’t expect to cure OUD during hospitalization
Withholding opioid will not cure patient’s OUD
Giving opioids will not worsen patient’s OUD
Initiate addiction treatment referral

## Slide 24
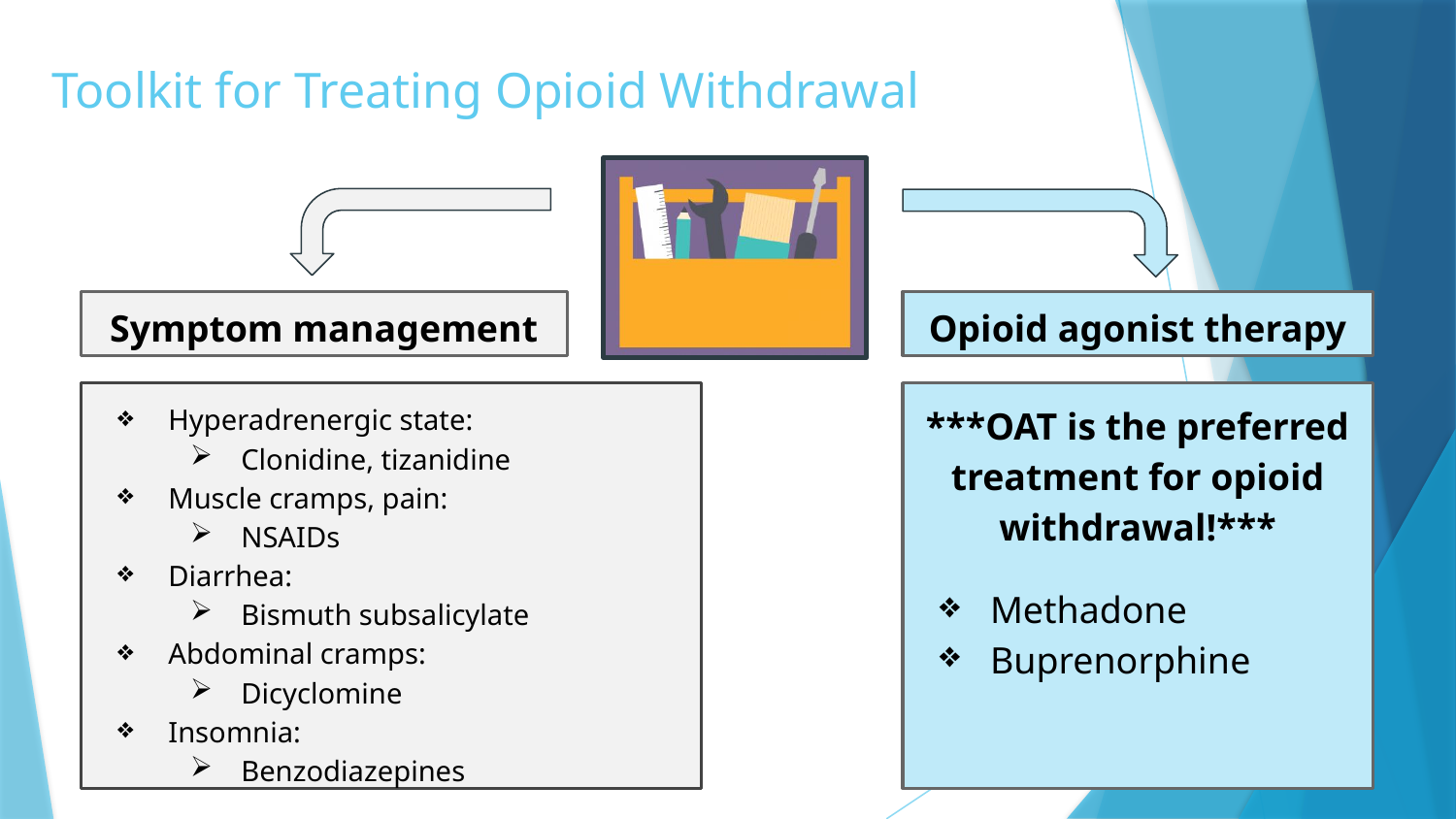

# Toolkit for Treating Opioid Withdrawal
Symptom management
Opioid agonist therapy
Hyperadrenergic state:
Clonidine, tizanidine
Muscle cramps, pain:
NSAIDs
Diarrhea:
Bismuth subsalicylate
Abdominal cramps:
Dicyclomine
Insomnia:
Benzodiazepines
***OAT is the preferred treatment for opioid withdrawal!***
Methadone
Buprenorphine

## Slide 25
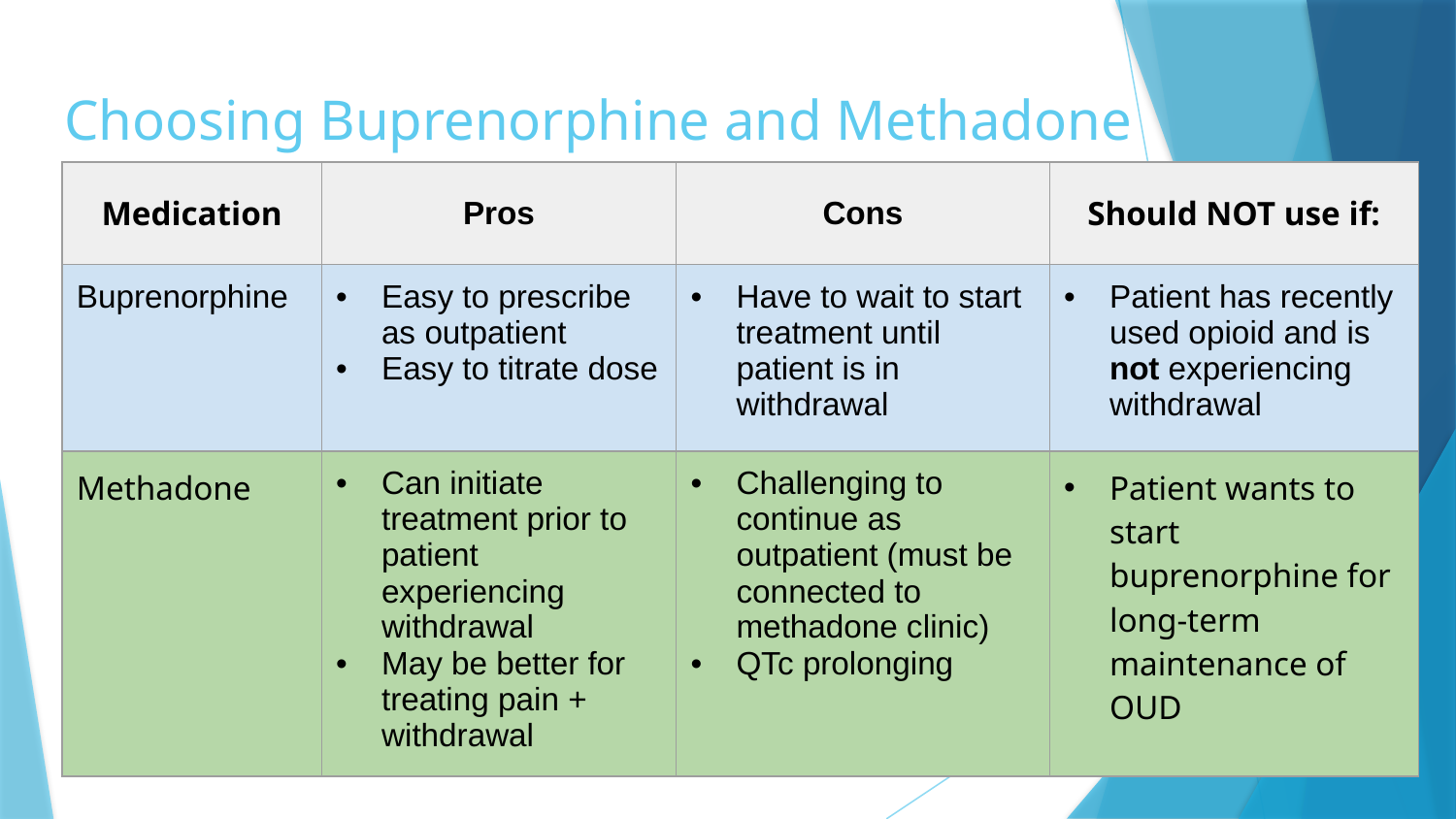

# Choosing Buprenorphine and Methadone
| Medication | Pros | Cons | Should NOT use if: |
| --- | --- | --- | --- |
| Buprenorphine | Easy to prescribe as outpatient Easy to titrate dose | Have to wait to start treatment until patient is in withdrawal | Patient has recently used opioid and is not experiencing withdrawal |
| Methadone | Can initiate treatment prior to patient experiencing withdrawal May be better for treating pain + withdrawal | Challenging to continue as outpatient (must be connected to methadone clinic) QTc prolonging | Patient wants to start buprenorphine for long-term maintenance of OUD |

## Slide 26
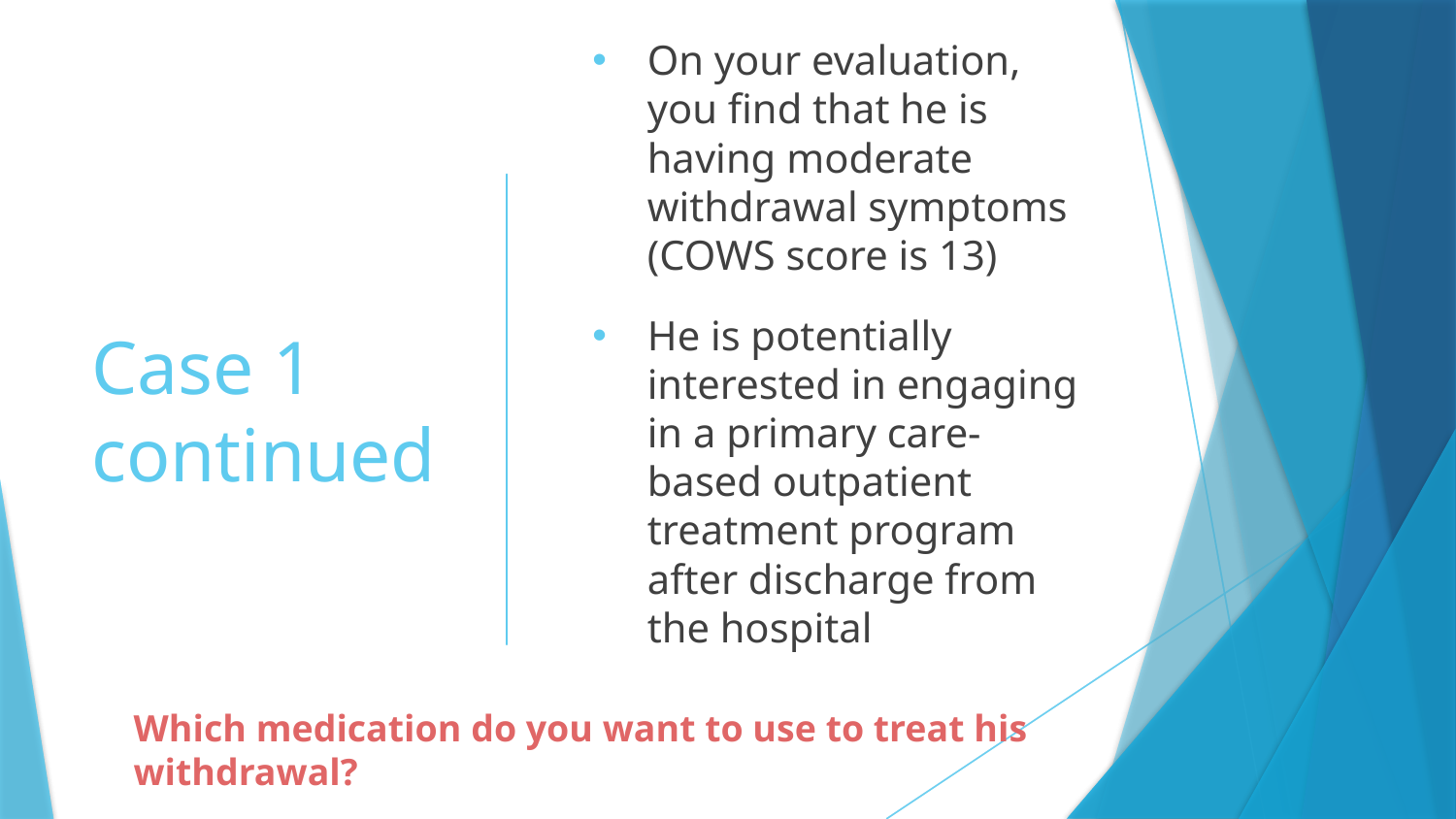

On your evaluation, you find that he is having moderate withdrawal symptoms (COWS score is 13)
He is potentially interested in engaging in a primary care-based outpatient treatment program after discharge from the hospital
# Case 1 continued
Which medication do you want to use to treat his withdrawal?

## Slide 27
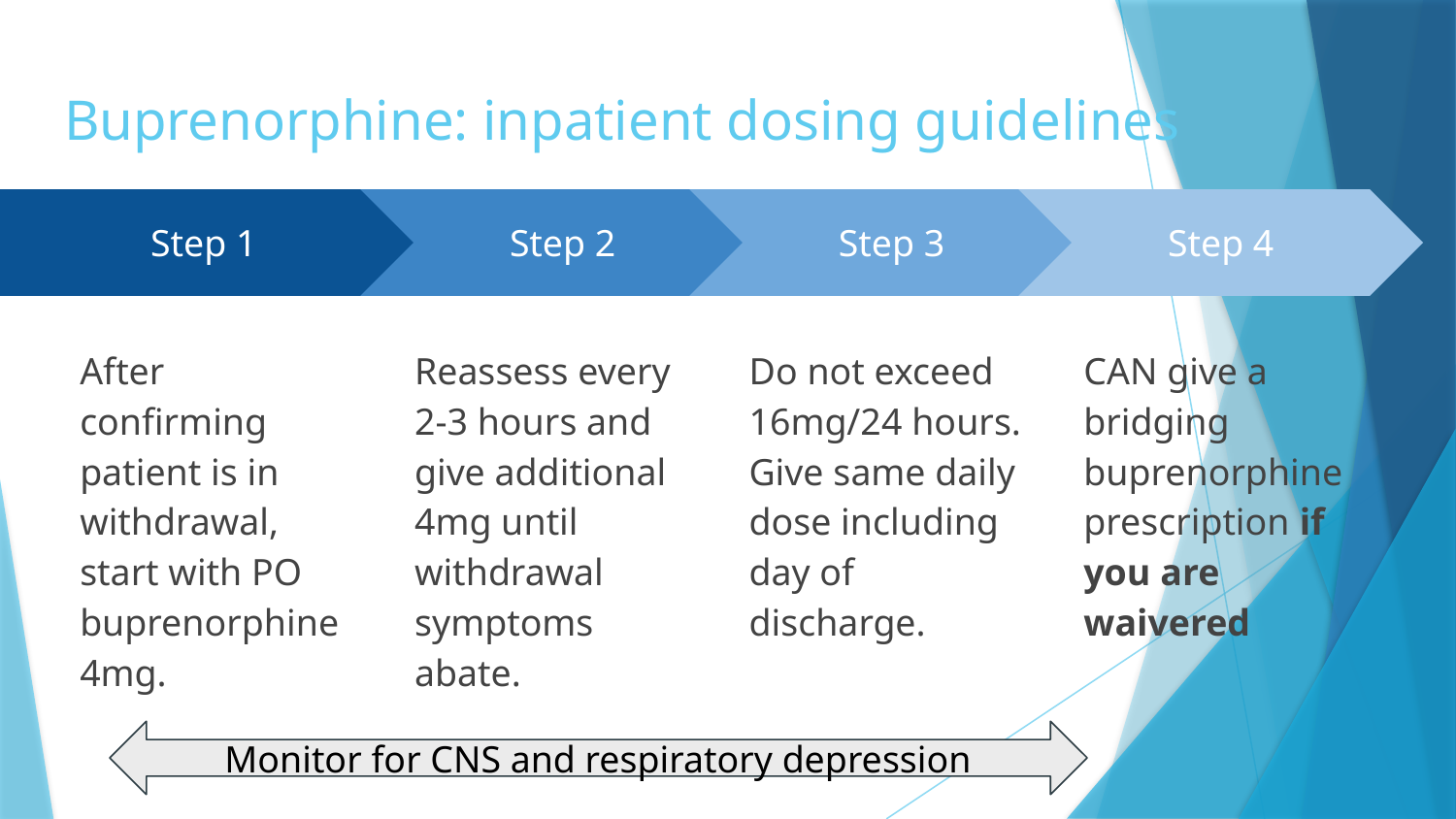

# Buprenorphine: inpatient dosing guidelines
Step 2
Reassess every 2-3 hours and give additional 4mg until withdrawal symptoms abate.
Step 3
Do not exceed 16mg/24 hours. Give same daily dose including day of discharge.
Step 4
CAN give a bridging buprenorphine prescription if you are waivered
Step 1
After confirming patient is in withdrawal, start with PO buprenorphine 4mg.
Monitor for CNS and respiratory depression

## Slide 28
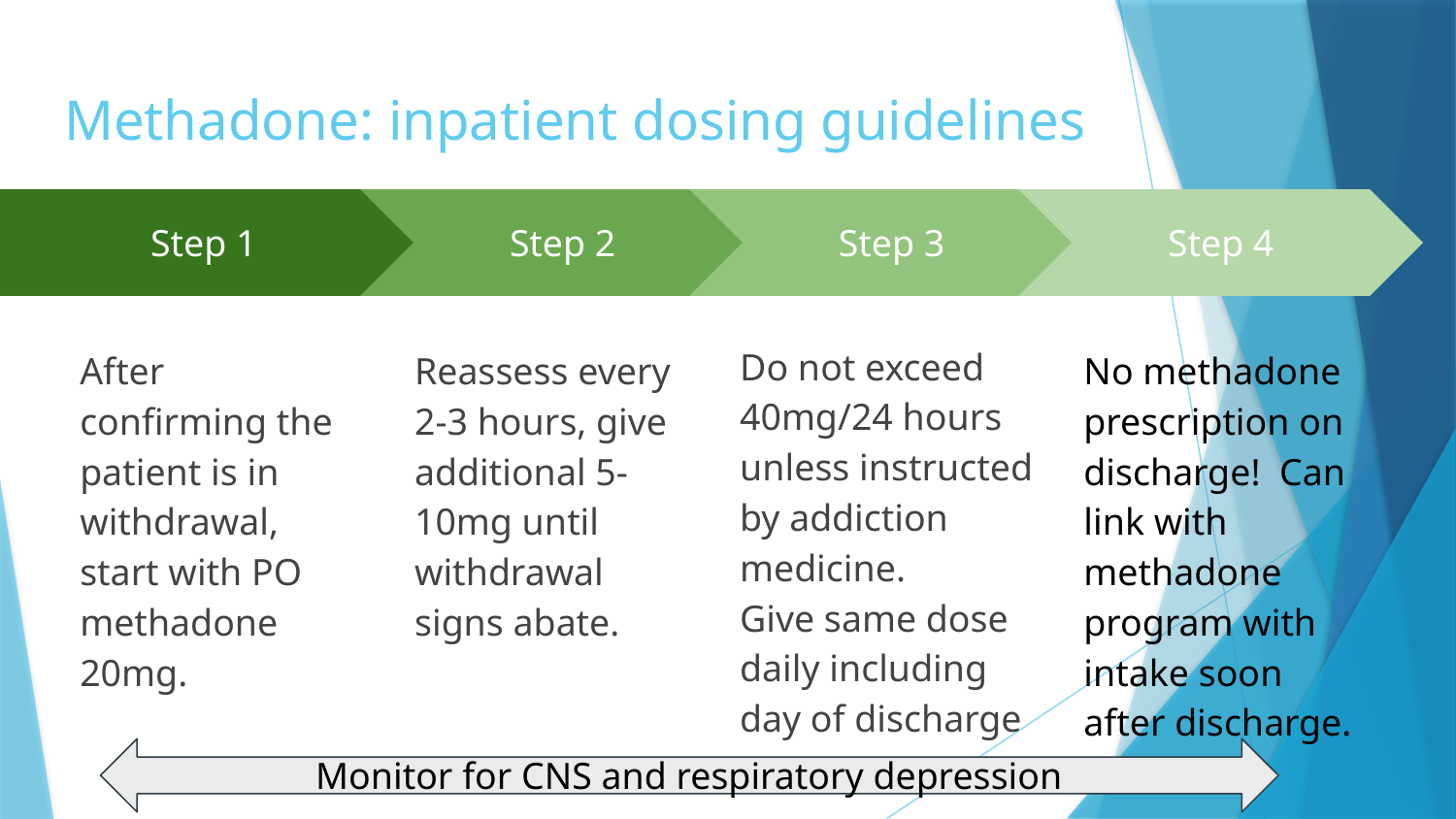

# Methadone: inpatient dosing guidelines
Step 2
Reassess every 2-3 hours, give additional 5-10mg until withdrawal signs abate.
Step 3
Do not exceed 40mg/24 hours unless instructed by addiction medicine.
Give same dose daily including day of discharge
Step 4
No methadone prescription on discharge! Can link with methadone program with intake soon after discharge.
Step 1
After confirming the patient is in withdrawal, start with PO methadone 20mg.
Monitor for CNS and respiratory depression

## Slide 29
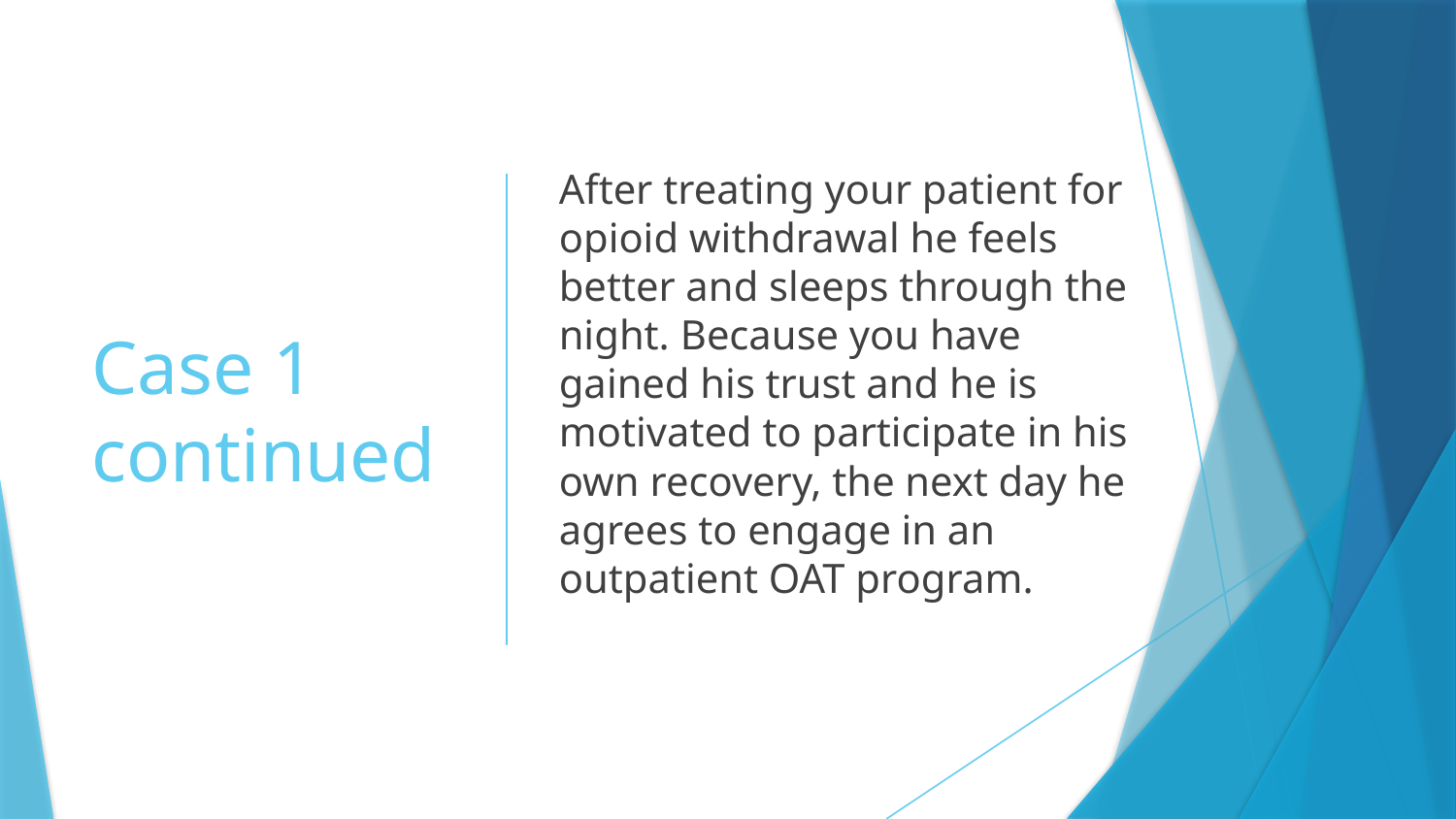

After treating your patient for opioid withdrawal he feels better and sleeps through the night. Because you have gained his trust and he is motivated to participate in his own recovery, the next day he agrees to engage in an outpatient OAT program.
# Case 1 continued

## Slide 30
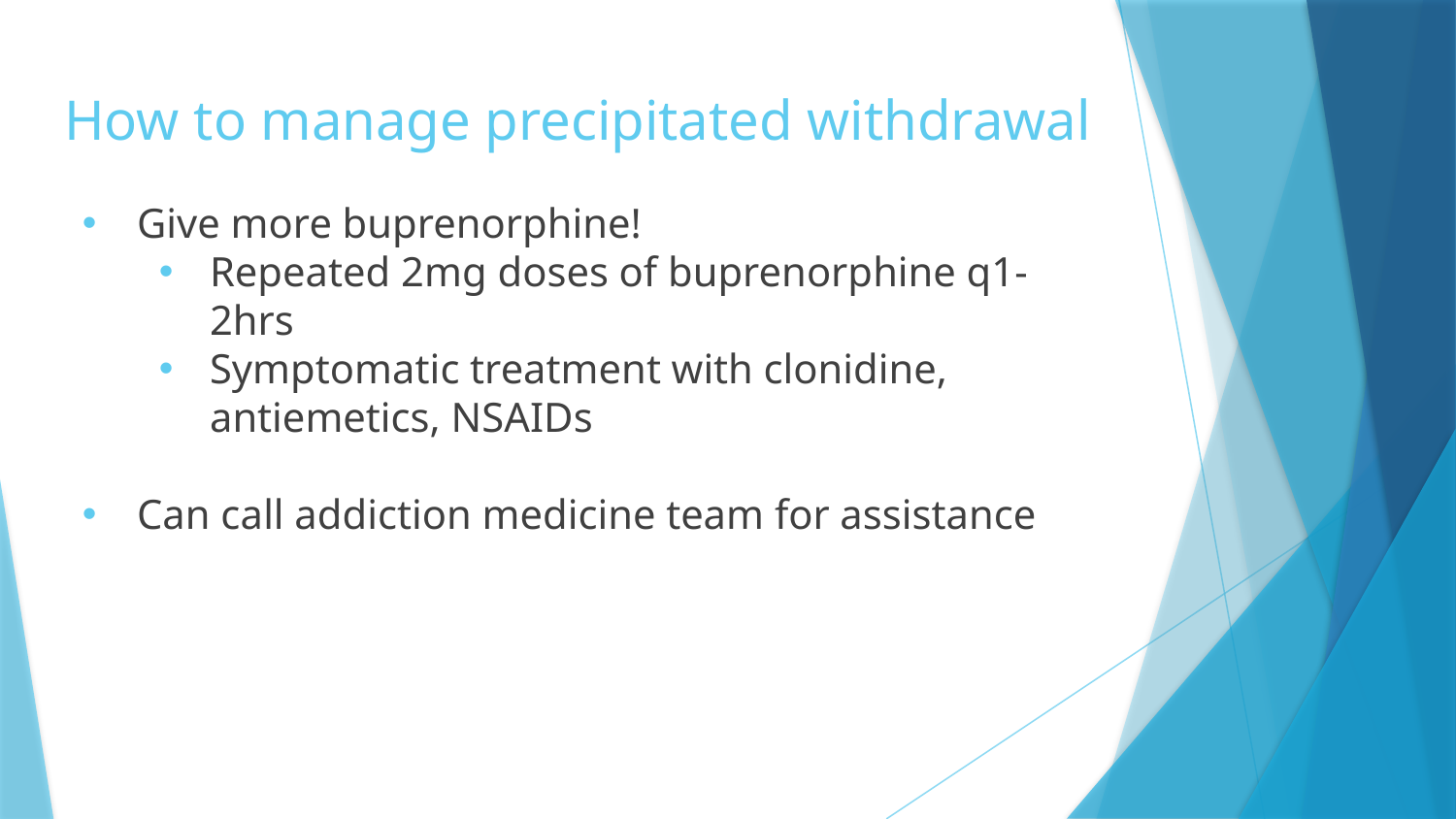

# How to manage precipitated withdrawal
Give more buprenorphine!
Repeated 2mg doses of buprenorphine q1-2hrs
Symptomatic treatment with clonidine, antiemetics, NSAIDs
Can call addiction medicine team for assistance

## Slide 31
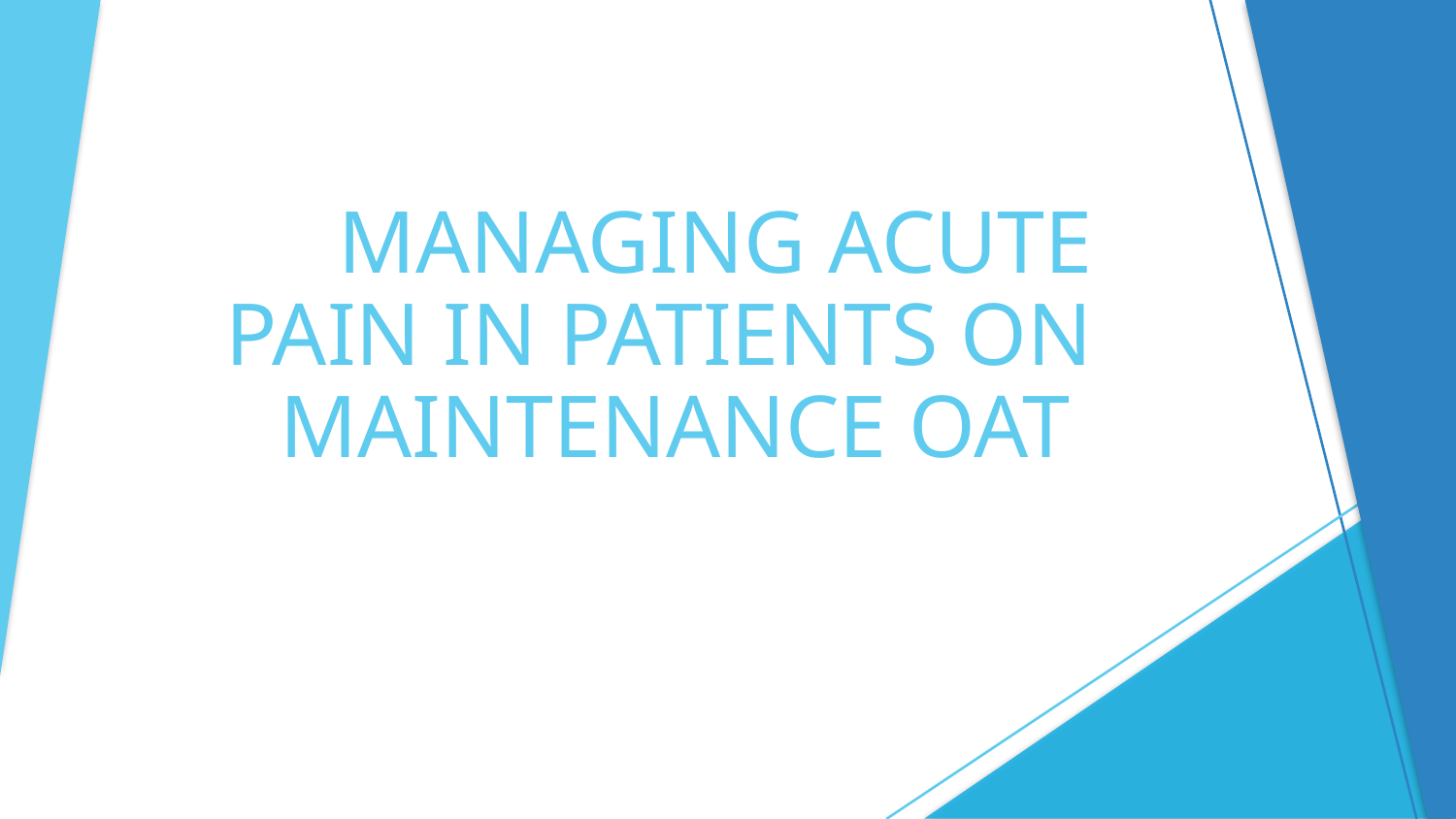

# MANAGING ACUTE PAIN IN PATIENTS ON MAINTENANCE OAT

## Slide 32
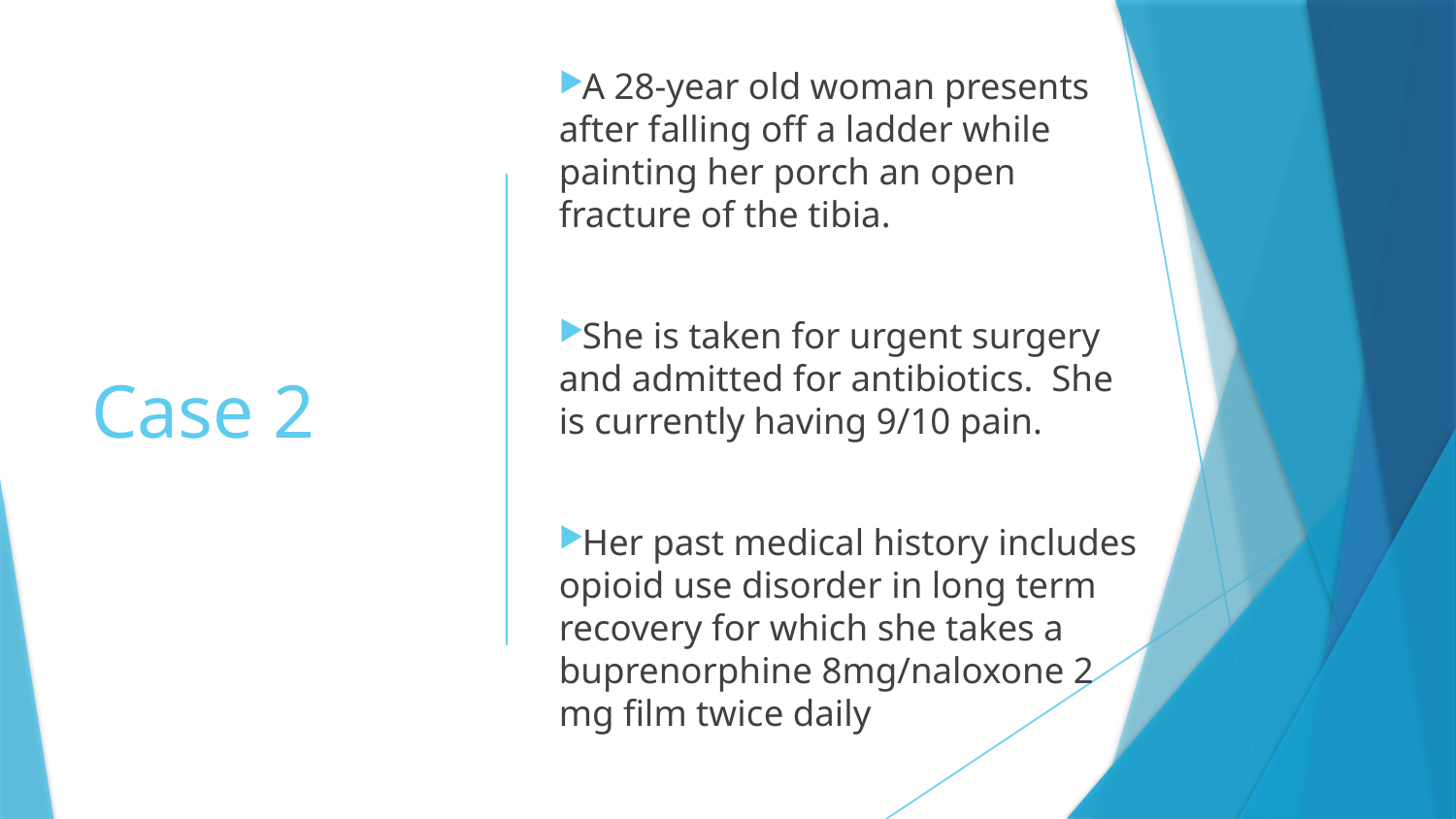

A 28-year old woman presents after falling off a ladder while painting her porch an open fracture of the tibia.
She is taken for urgent surgery and admitted for antibiotics. She is currently having 9/10 pain.
Her past medical history includes opioid use disorder in long term recovery for which she takes a buprenorphine 8mg/naloxone 2 mg film twice daily
# Case 2

## Slide 33
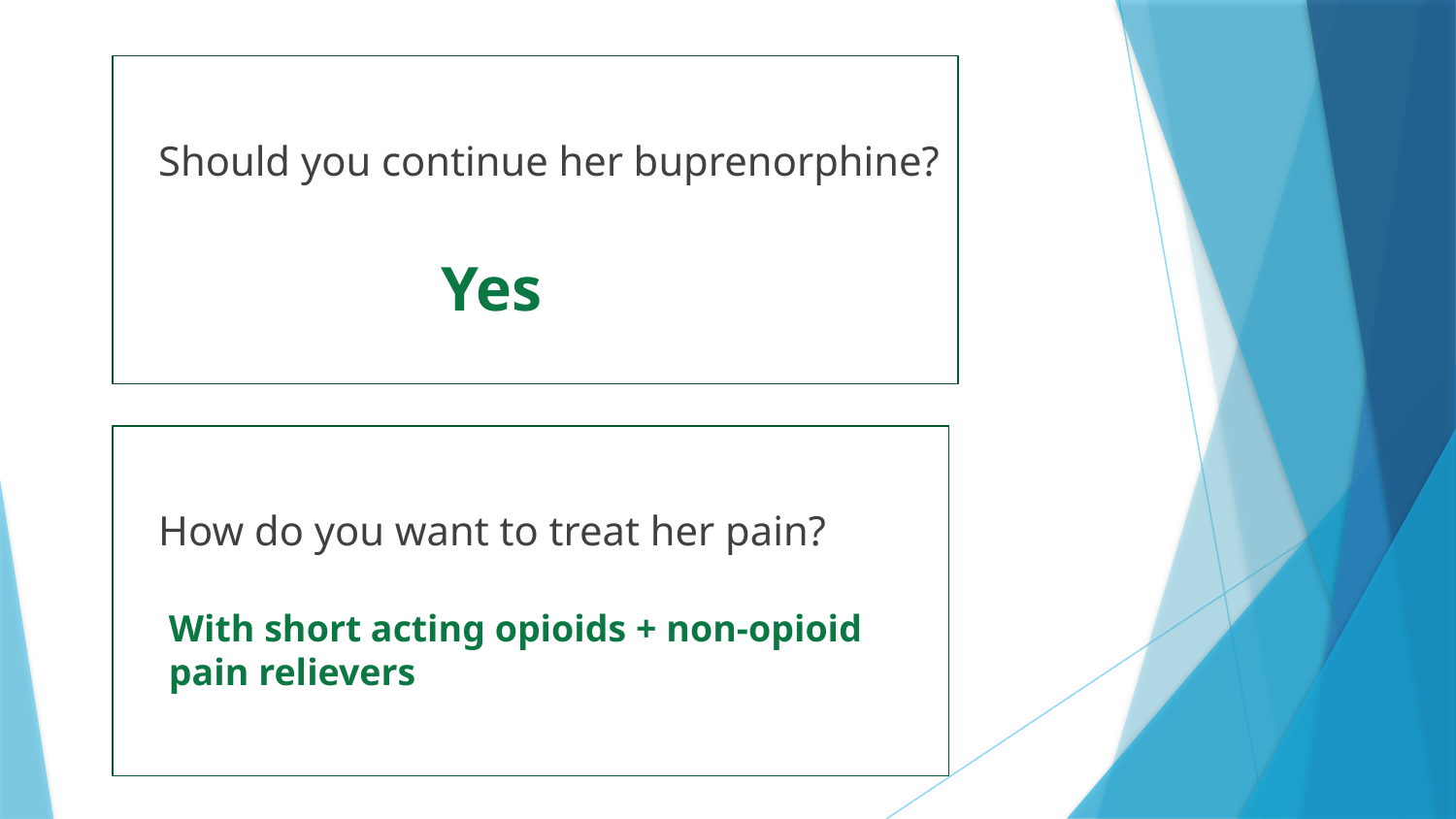

Should you continue her buprenorphine?
Yes
 How do you want to treat her pain?
With short acting opioids + non-opioid pain relievers

## Slide 34
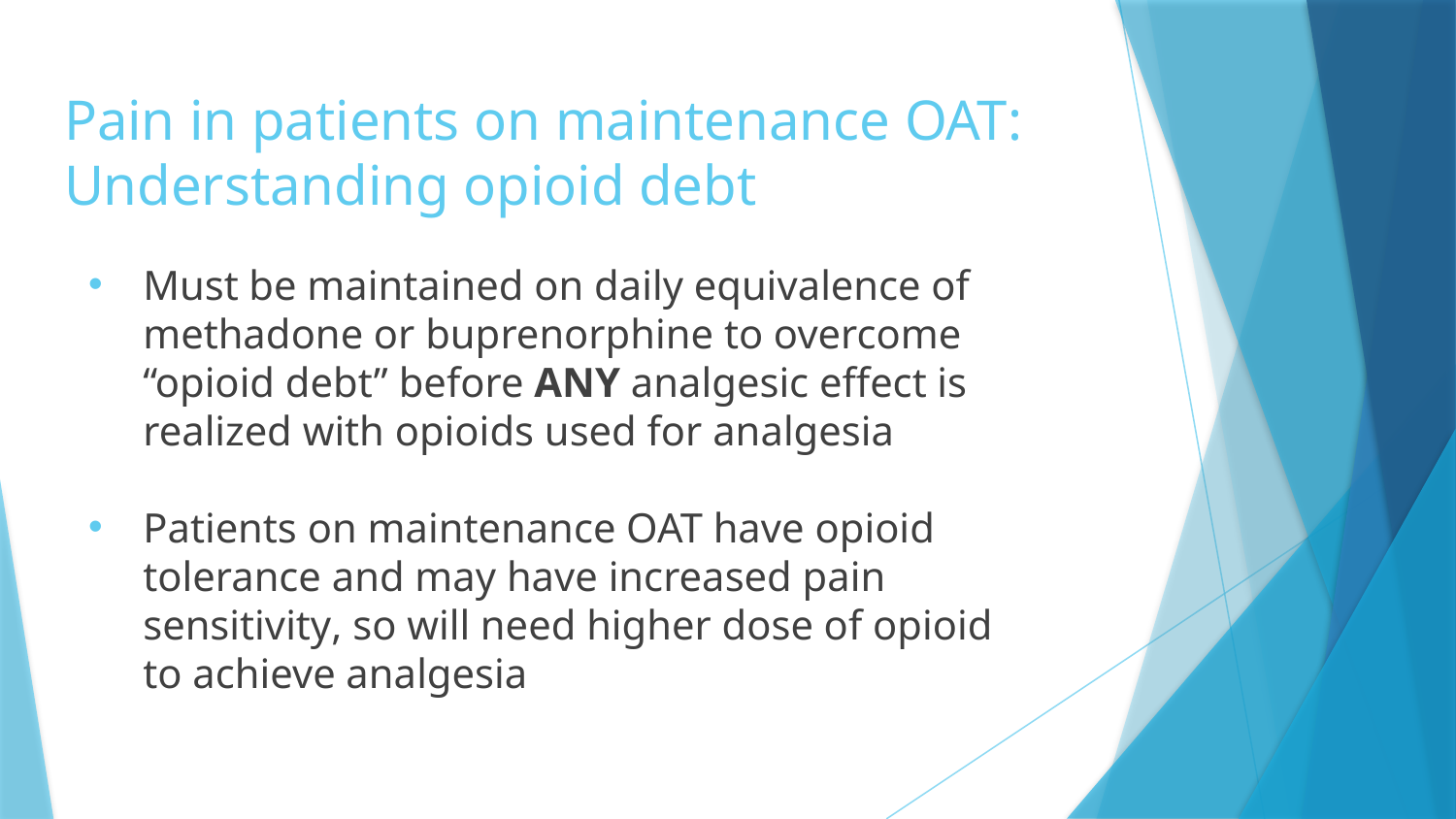

# Pain in patients on maintenance OAT: Understanding opioid debt
Must be maintained on daily equivalence of methadone or buprenorphine to overcome “opioid debt” before ANY analgesic effect is realized with opioids used for analgesia
Patients on maintenance OAT have opioid tolerance and may have increased pain sensitivity, so will need higher dose of opioid to achieve analgesia

## Slide 35
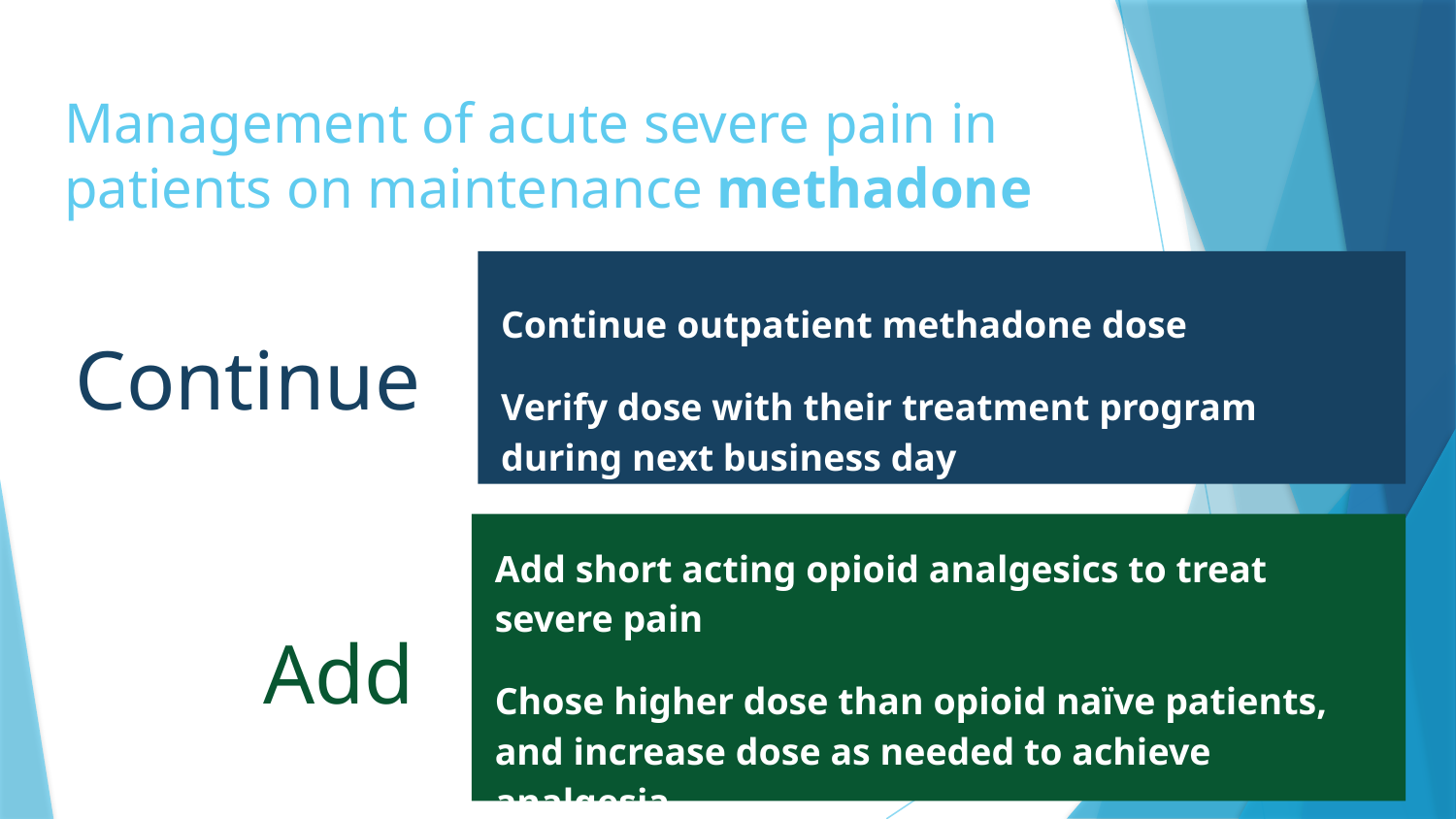

# Management of acute severe pain in patients on maintenance methadone
Continue
Continue outpatient methadone dose
Verify dose with their treatment program during next business day
Add
Add short acting opioid analgesics to treat severe pain
Chose higher dose than opioid naïve patients, and increase dose as needed to achieve analgesia

## Slide 36
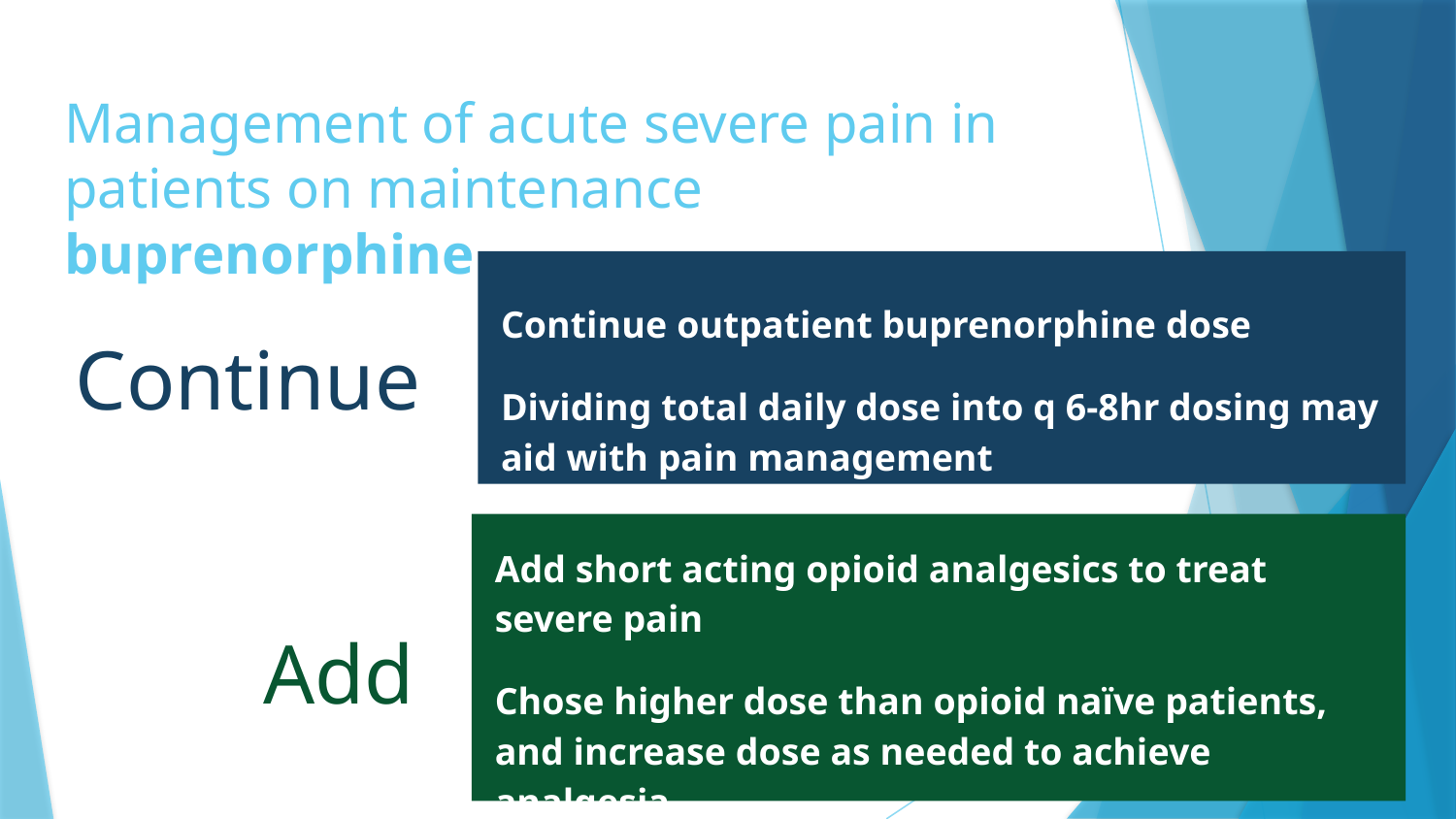

# Management of acute severe pain in patients on maintenance buprenorphine
Continue
Continue outpatient buprenorphine dose
Dividing total daily dose into q 6-8hr dosing may aid with pain management
Add
Add short acting opioid analgesics to treat severe pain
Chose higher dose than opioid naïve patients, and increase dose as needed to achieve analgesia

## Slide 37
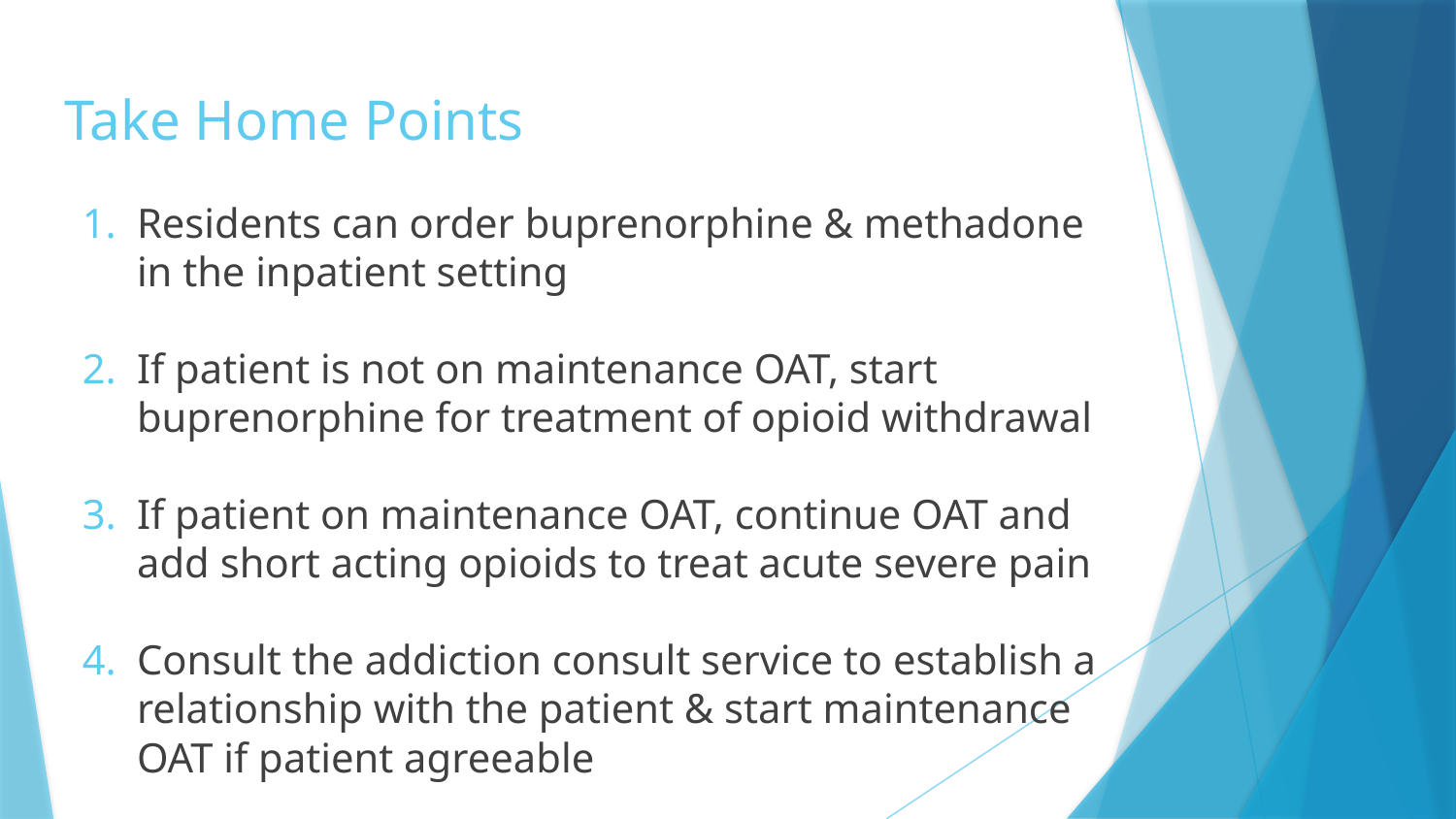

# Take Home Points
Residents can order buprenorphine & methadone in the inpatient setting
If patient is not on maintenance OAT, start buprenorphine for treatment of opioid withdrawal
If patient on maintenance OAT, continue OAT and add short acting opioids to treat acute severe pain
Consult the addiction consult service to establish a relationship with the patient & start maintenance OAT if patient agreeable
