# Supplementary material for: Treating Withdrawal and Pain in Inpatients With Opioid Use Disorder: A Brief Educational Intervention for Internal Medicine Residents
Source: MedEdPORTAL. 2021 Mar 10;17:11123. doi: 10.15766/mep_2374-8265.11123 (PMC7970646; doi:10.15766/mep_2374-8265.11123)
Supplement: Supplementary file 1 — Presentation Materials.pptxPre- and Postsurvey.docx [file mep_2374-8265.11123-s001.zip › B. Pre- and Postsurvey.docx]

**Treating Acute Opioid Withdrawal and Acute Pain in Patients with Opioid Use Disorder Survey**

You are being asked to participate in a survey as part of an educational intervention on the inpatient management of opioid withdrawal and acute pain in patients on opioid agonist therapy. The survey below will take 5 minutes on average. Your participation in this survey is entirely optional. Participating, or not participating, will have no bearing on your standing within the residency program, and the results will be kept anonymous. Thank you for your participation!

**Survey ID:**

Please enter the last letter of your first name, the first letter of your last name, and the last 4 numbers of your phone number. (Example: Jane Doe: ED1586)

__________________________________

Is this your pre-survey (first time answering this survey) or 30-day post-survey (second time)?

- Pre-Survey
- Post-Survey

**CONFIDENCE:** The following questions are regarding your level of confidence in each of the following situations.

| How confident are you about... | 1  Not at all  confident | 2  Slightly  confident | 3 Somewhat  confident | 4  Very  confident | 5  Extremely  confident |
| --- | --- | --- | --- | --- | --- |
| …asking patients with opioid use disorder about their opioid use? |  |  |  |  |  |
| …choosing between methadone  or buprenorphine when managing acute opioid withdrawal in the inpatient setting? |  |  |  |  |  |
| …ordering methadone for the inpatient management of acute opioid withdrawal? |  |  |  |  |  |
| …ordering buprenorphine for the  inpatient management of acute  opioid withdrawal? |  |  |  |  |  |
| …dosing opioid to treat acute pain in patients on maintenance buprenorphine or methadone? |  |  |  |  |  |

**SELF-REPORTED PRACTICE:** The following question is regarding your use of opioid agonist therapy (e.g. methadone, buprenorphine) to treat acute opioid withdrawal in the inpatient

setting.

During your last inpatient floors month, did you treat any patients with opioid use disorder who were experiencing withdrawal symptoms with opioid agonist therapy (either buprenorphine or methadone)?

- Yes
- No

**KNOWLEDGE:** The following questions are regarding your knowledge related to treating acute opioid withdrawal and acute pain in patients on opioid agonist therapy in the inpatient setting. Correct answers are in **bold**.

1. A 32-year old woman is admitted to your inpatient medicine service with severe right upper extremity cellulitis.

She is actively using intravenous heroin (0.5 grams [10 bags] per day) and is not currently in addiction treatment. Eight hours after admission she begins to experience moderate opioid withdrawal. The best initial treatment for her opioid withdrawal during her inpatient hospital stay is which of the following:

- **Buprenorphine 4mg PO**
- Buprenorphine 16mg PO
- Clonazepam 0.5mg PO
- Clonidine 0.1mg PO

2. A 45-year old man with daily use of intravenous heroin who was admitted this morning for severe opioid

withdrawal was given 4mg buprenorphine around 5pm. You are on nightfloat when the nurse calls you at 8pm due to improved, but persistent tachycardia, diaphoresis, nausea, abdominal pain, and myalgias. The best next step for treatment is:

- Clonidine, loperamide, acetaminophen, NSAIDs for symptomatic relief
- Give methadone 20mg for withdrawal, as buprenorphine was not effective
- **Give another 4mg buprenorphine for persistent withdrawal and reassess in 2-3 hours**
- Give high doses of opioids to treat precipitated opioid withdrawal

3. A 30-year old man with daily oxycodone use for the past 12 months develops acute nausea and vomiting after his first dose of buprenorphine. What is your most likely diagnosis?

- Buprenorphine allergy
- Viral syndrome
- Acute opioid intoxication
- **Precipitated opioid withdrawal**

4. A 58-year old woman is in severe pain secondary to acute gallstone pancreatitis. She is on methadone

maintenance treatment with a dose of 80mg per day. What is the best treatment option for her acute pain?

- Discontinue methadone and treat with scheduled IV morphine every 4 hours
- Decrease her methadone to 20mg with PRN IV morphine every 6-8 hours
- **Maintain methadone at 80mg daily with PRN IV morphine every 4-6 hours**
- Increase her methadone to 110mg every 24 hours to treat her pain

5. Which of the following statements is TRUE regarding prescribing limitations for initiating methadone and

buprenorphine by non-waivered internal medicine residents in the treatment of opioid use disorder?

- Residents can initiate methadone in both the inpatient and outpatient setting
- **Residents can initiate both methadone and buprenorphine in the inpatient setting**
- Residents can initiate buprenorphine, but not methadone, in the inpatient setting
- Residents can initiate methadone, but not buprenorphine, in the inpatient setting
