# Supplementary material for: Treating Withdrawal and Pain in Inpatients With Opioid Use Disorder: A Brief Educational Intervention for Internal Medicine Residents
Source: MedEdPORTAL. 2021 Mar 10;17:11123. doi: 10.15766/mep_2374-8265.11123 (PMC7970646; doi:10.15766/mep_2374-8265.11123)
Supplement: Supplementary file 1 — Presentation Materials.pptxPre- and Postsurvey.docx [file mep_2374-8265.11123-s001.zip › _Educational_Summary_Report_11123.pdf]

MedEdPORTAL® | The AAMC Journal of Teaching and Learning Resources

Original Publication Open Access

Treating Withdrawal and Pain in Inpatients With Opioid Use Disorder: A Brief Educational Intervention for Internal Medicine Residents

Ayako Wendy Fujita, MD, Anna LaRosa, MD, Andrea Carter, MD, MS*

*Corresponding author: carterae@upmc.edu

Abstract

Introduction: Despite the effectiveness of opioid agonist therapy (OAT) for treating patients with opioid use disorder (OUD), insufficient education remains a barrier to prescribing. Internal medicine (IM) residents are optimally positioned to facilitate use of OAT, especially in the inpatient setting. We implemented an educational intervention aimed at increasing IM residents' knowledge and confidence regarding prescribing OAT to inpatients with OUD. **Methods:** We created a 35-minute, case-based presentation highlighting the management of opioid withdrawal using OAT and treating pain in inpatients on maintenance OAT. It was presented to IM residents beginning their general medicine ward rotations from November 2019 through January 2020. We developed a survey to measure participants' knowledge (mean number of questions correct out of five) and confidence (mean Likert-scale score, 1 = *Not at all confident,* 5 = *Extremely confident,* on each of five items) regarding prescribing OAT in the inpatient setting. We compared knowledge and confidence before versus 1 month after the intervention using paired Student *t* tests, with $p < .05$ indicating significance. **Results:** Of 103 unique residents completing ward rotations, 29 (28%) completed both the pre- and 1-month postsurveys and were included in the analysis. The mean number of knowledge questions correct increased from 3.1 pre- to 4.3 postintervention, and mean confidence scores increased from below 2 pre- to over 3 postintervention in four of five items ($ps < .001$). **Discussion:** A brief, generalizable, educational intervention significantly increased residents' knowledge of and confidence in prescribing OAT in inpatients with OUD.

Keywords
Opioid Use Disorder, Opioid Withdrawal, Internal Medicine, Hospital Medicine, Substance Abuse/Addiction, Clinical Teaching/Bedside Teaching, Opioids, Addiction, Pain

Educational Objectives

At the end of this intervention, learners will be able to:

1. Choose between methadone and buprenorphine for treatment of inpatient opioid withdrawal.
2. Describe initial dosing regimens of methadone and buprenorphine for treatment of inpatient opioid withdrawal.
3. Identify and treat precipitated withdrawal from buprenorphine initiation.
4. Treat severe, acute pain using short-acting opioids in inpatients on maintenance opioid agonist therapy.

Citation:
Fujita AW, LaRosa A, Carter A. Treating withdrawal and pain in inpatients with opioid use disorder: a brief educational intervention for internal medicine residents. *MedEdPORTAL.* 2021;17:11123.
https://doi.org/10.15766/mep_2374-8265.11123

Introduction

Patients with opioid use disorder (OUD) and injection drug use have high rates of readmission, recurrent infections, and mortality, yet despite this, they often receive suboptimal addiction care.[1] Opioid agonist therapy (OAT), including methadone and buprenorphine, is the standard of care for treating inpatient opioid withdrawal symptoms and may help patients with OUD engage in longer-term care.[2-5] Initiation of buprenorphine in the inpatient setting has been shown to be effective for engaging medically hospitalized patients who are not seeking addiction treatment and reduces illicit opioid use 6 months after hospitalization.[4] However, delivery of OAT in the inpatient setting is infrequent and varies widely, which has led to calls for educational interventions to promote hospital-based OAT delivery.[6]

Internal medicine (IM) residents, as the primary physicians caring for many inpatients with OUD, are optimally positioned to facilitate inpatient use of OAT. Although residents who are

Copyright © 2021 Fujita et al. This is an open-access publication distributed under the terms of the Creative Commons Attribution license.

not waiver trained cannot prescribe OAT on discharge, inpatient clinicians are exempt from these regulations and may provide both methadone and buprenorphine for maintenance and withdrawal treatment in the inpatient setting.[2] Despite this, education about treatment of OUD remains inadequate in medical training.[7] Although there are evidence-based guidelines on substance use screening, assessment, and management, integration of this research into residency curricula remains challenging. In one survey, 77% of residency program directors reported that residents frequently cared for patients with OUD, yet only 23% reported spending 12 or more hours of curricular time on addiction.[8] Furthermore, most prior educational interventions on OAT are based in outpatient settings[9-11] or the emergency department[12] rather than the inpatient setting. There have been no prior studies of educational interventions aimed at broadly teaching all IM residents within a program to manage OAT in the inpatient setting.

We developed, implemented, and evaluated an educational intervention for IM residents on management of inpatient opioid withdrawal with OAT and management of acute pain in patients on maintenance OAT. Our aims were to increase participants' knowledge, confidence, and self-reported frequency of prescribing OAT in the inpatient setting. The IM residency program is the largest program in our hospital system, with over 200 trainees. Although there is growing attention to OUD within the program, including an addiction medicine elective rotation, there is currently no formal curriculum addressing OAT in the inpatient setting. Therefore, the IM residency program was an ideal venue to provide education on appropriate utilization of OAT for patients with OUD presenting with opioid withdrawal or acute pain. Given the lack of postgraduate education on prescribing OAT nationally, it is important to evaluate and disseminate this educational intervention, as it could inform similar curricula throughout other residency training programs.

Methods

We developed, implemented, and evaluated an educational intervention within an urban academic IM residency program. To evaluate the effectiveness of the intervention, we developed a survey to measure participants' knowledge, confidence, and self-reported frequency of prescribing OAT in the patient setting pre- compared to postintervention. The intervention was deemed not research by the University of Pittsburgh Institutional Review Board (ID 191004).

Setting and Participants

IM residents at all training levels scheduled for an inpatient general medicine rotation from November 2019 through January 2020 at two tertiary care academic hospital sites within the University of Pittsburgh Medical Center IM residency program were required to participate in the intervention. Two IM chief medical residents (CMRs) delivered the content. Both CMRs participated in the Chief Resident Immersion Training (CRIT) program in addiction medicine, a 4-day immersion training that equipped rising CMRs with skills to manage and teach about addiction.[7] Our presentation materials were adapted with permission from resources and citations provided at the CRIT program. Funding for attendance was provided by the CRIT program as well as by our Division of General Internal Medicine.

Although the CRIT program was open to all rising IM or family medicine chief residents and their faculty mentors, it may not be available to all who use this intervention in the future. We have included ample text in the presentation material slides and presenter notes. Additionally, we recommend that future presenters, especially if they do not have personal expertise in OUD, review the presentation materials with local content experts to ensure concordance with local practices, particularly the availability and dosing of medications for OUD in the inpatient setting.

Educational Intervention

We developed a case-based presentation highlighting the management of inpatient opioid withdrawal and acute pain in inpatients on maintenance OAT (Appendix A). Learning points included choosing between methadone and buprenorphine for treatment of withdrawal, initial dosing of methadone and buprenorphine, identification and treatment of precipitated withdrawal, and managing short-acting opioids in patients on maintenance OAT with acute pain. The content was reviewed with local experts to ensure concordance with local practices. The presentation was delivered monthly by a CMR during the preexisting 1-hour rotation orientation, which consisted of a 25-minute abbreviated orientation including rotation-specific information, daily schedules, and announcements followed by this 35-minute educational intervention. A conference room with a laptop, projector, and screen was utilized for each session.

Outcomes Ascertainment

We developed a survey to measure knowledge, confidence, and self-reported prescribing (Appendix B). We delivered the survey via email to residents participating in the educational intervention each month 3 days preintervention and again 1 month after the intervention. The survey was developed with local experts in addiction medicine and medical education to ensure appropriateness, clarity, and completeness and was revised after pretesting with CMRs and faculty. Knowledge was

Copyright © 2021 Fujita et al. This is an open-access publication distributed under the terms of the Creative Commons Attribution license.

measured with five multiple-choice questions assessing (1) initial dosing of OAT for treatment of withdrawal, (2) treatment of persistent withdrawal symptoms after buprenorphine initiation, (3) identification of precipitated withdrawal, (4) treatment of acute pain in a patient on methadone maintenance, and (5) regulations regarding OAT. Confidence was measured with five 5-point Likert-scale items (1 = *Not at all confident*, 5 = *Extremely confident*) regarding confidence with (1) asking patients about opioid use, (2) choosing between methadone and buprenorphine for treatment of withdrawal, ordering (3) methadone and (4) buprenorphine for treatment of withdrawal, and (5) dosing opioids to treat acute pain in patients on OAT. To assess prescribing practices, a single item asked if respondents had prescribed OAT to treat opioid withdrawal during their most recent ward rotation.

Data Analysis

We included residents who completed both the pre- and 1-month postsurveys in the analysis. We compared the mean number of knowledge questions answered correctly per resident, the proportion of residents answering each knowledge question correctly, and mean Likert-scale response for each confidence item pre- versus postintervention using paired Student *t* tests. We compared the proportion of residents who had prescribed OAT to treat withdrawal during their previous ward rotation pre- versus postintervention using a chi-square test. A *p* value less than .05 was considered significant. All analyses were performed using Stata SE 15.1 (StataCorp).

Results

Of 103 unique residents completing ward rotations from November 2019 through January 2020, 29 (28%) completed both the pre- and 1-month postsurveys and were included in the analysis.

The mean number of knowledge questions correct out of five increased from 3.1 pre- to 4.3 postintervention ($p < .001$). Significantly more residents correctly answered knowledge questions postintervention compared to preintervention in four of the five questions, including questions regarding the initial treatment of inpatient withdrawal ($p < .001$), treatment of persistent withdrawal after initiation of buprenorphine ($p = .03$), treatment of acute pain in a patient with OUD on methadone maintenance ($p = .023$), and regulations regarding OAT treatment ($p < .001$). The proportion of residents able to identify precipitated withdrawal was high (93%) both pre- and postintervention ($p = 1$; see Figure 1).

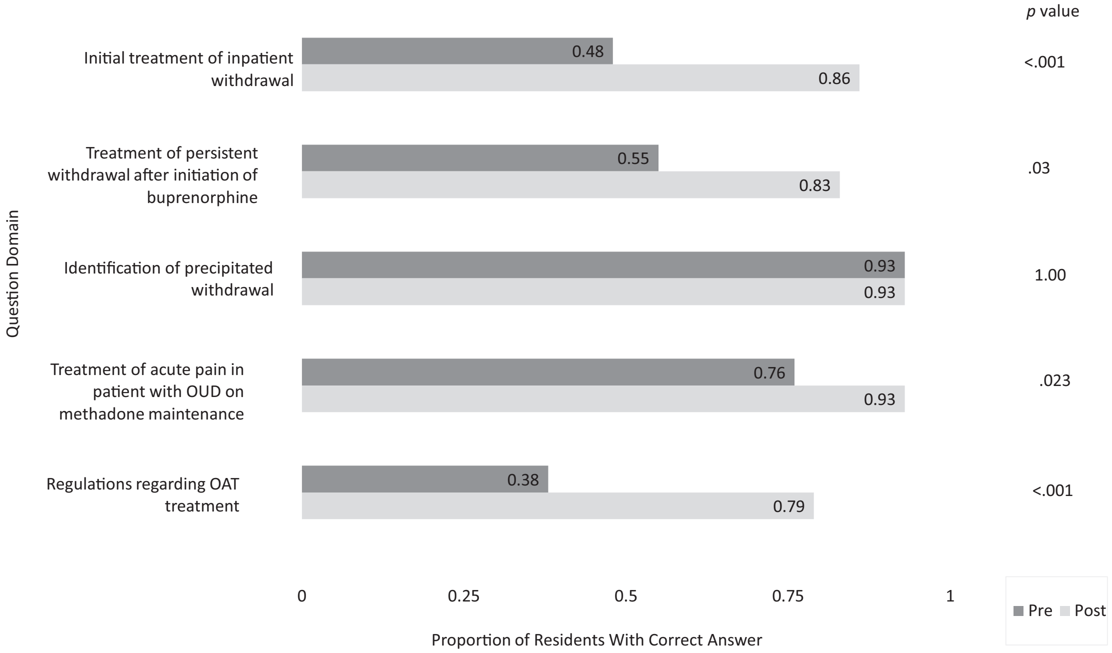

Figure 1. Proportion of residents with correct answers to each knowledge question regarding inpatient opioid agonist therapy (OAT) pre- and postintervention. Internal medicine residents scheduled for a general medicine rotation at a large, urban, academic medical center were required to participate in a brief education intervention highlighting the basic management of inpatient opioid withdrawal and acute pain in inpatients on maintenance OAT. A survey containing five multiple-choice knowledge questions was administered before and 1 month after the intervention. Respondents completing both surveys were included in the analysis, and the proportion of residents with the correct answer to each question was compared pre- versus postintervention using paired Student *t* tests, with $p < .05$ considered significant. Significantly more residents correctly answered each knowledge question post- compared to preintervention in four of five questions. OUD, opioid use disorder.

Copyright © 2021 Fujita et al. This is an open-access publication distributed under the terms of the Creative Commons Attribution license.

Mean confidence scores increased from below 2 pre- to over 3 postintervention in four domains, including choosing between methadone and buprenorphine for treatment of withdrawal, ordering methadone for treatment of withdrawal, ordering buprenorphine for treatment of withdrawal, and dosing opioids to treat acute pain in patients on OAT ($p < .001$). Mean confidence remained over 3 for asking patients about opioid use ($p = .17$; see Figure 2).

Of the 29 residents included, 11 (38%) indicated that they had treated a patient experiencing opioid withdrawal with OAT during their most recent floor month postintervention compared to five (27%) preintervention ($p = .078$).

Discussion

A CMR-led intervention during preexisting curricular time significantly increased residents' knowledge and confidence about inpatient management of opioid withdrawal and pain in patients on maintenance OAT. Resident knowledge and confidence each increased in four out of five items pre- compared to postintervention, with the remaining items having high scores both pre- and postintervention, suggesting a ceiling effect. There was no difference in self-reported prescribing of OAT for withdrawal.

IM residents, as physicians caring for many inpatients with OUD, should be educated universally. By delivering recurrent sessions monthly in a required rotation, we reached all residents in the program over a year. Prior educational interventions such as an addiction medicine elective[13] or an optional longitudinal curriculum[10] have reached a minority of residents.

Our educational intervention may be more practically implemented than previously published ones. First, we utilized CMRs to teach. CMRs play a pivotal role in educating trainees and often become change agents in future leadership roles.[7] They may also have more time availability than faculty as used in prior interventions.[9,10,13] Additionally, the timing during preexisting orientation sessions omitted the need for additional curricular time and empowered residents to immediately apply

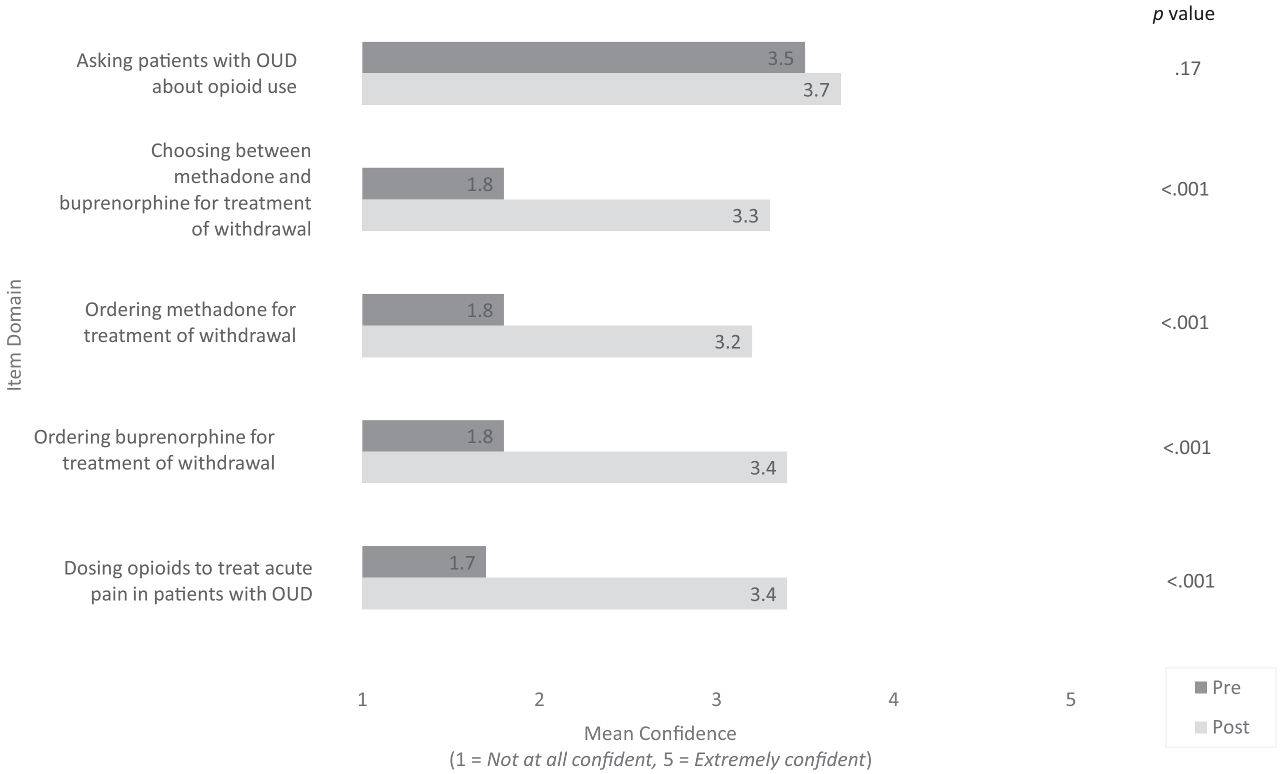

Figure 2. Residents' mean confidence for each confidence item regarding inpatient opioid agonist therapy (OAT) pre- compared to postintervention. Internal medicine residents scheduled for a general medicine rotation at a large, urban, academic medical center were required to participate in a brief education intervention highlighting the basic management of inpatient opioid withdrawal and acute pain in inpatients on maintenance OAT. A survey containing five Likert-scale confidence items (1 = *Not at all confident,* 5 = *Extremely confident*) was administered before and 1 month after the intervention. Respondents completing both surveys were included in the analysis, and the mean Likert-scale response on each item was compared pre- versus postintervention using paired Student *t* tests, with $p < .05$ considered significant. Mean confidence increased significantly in four out of five items post- compared to preintervention. OUD, opioid use disorder.

Copyright © 2021 Fujita et al. This is an open-access publication distributed under the terms of the Creative Commons Attribution license.

the knowledge and skills learned. A prior study describing a safe opioid prescribing education intervention found that a lecture followed immediately by an objective structured clinical examination had the greatest impact on residents' knowledge and confidence because learners had the opportunity to practice and solidify their new skills, which allowed for rapid integration.[9] By delivering our content on the first day of an IM floor rotation, we achieved similar benefits: Residents had the opportunity to apply and integrate these skills without additional resources such as standardized patients. Given the positive reception and improvements observed in knowledge and confidence, we continued delivering this intervention monthly past our evaluation period until a full academic year had passed and all IM residents in our program had received the content. We plan to continue delivering the intervention in future academic years during our intern orientation sessions.

Our study has limitations. We implemented our intervention within a single residency program, so our findings may not be generalizable to other programs. While we involved content experts in the development of our survey, it did not undergo further reliability or validity testing. The lack of a control group makes it difficult to fully attribute improvements to our intervention. We could not assess for differences between survey responders and nonresponders, and the reduced survey participation may have biased findings if those who were more invested in the curriculum were more likely to complete the survey. Self-reported prescribing may have been subject to recall bias, and lack of prescribing may have been due to not encountering an appropriate patient scenario. Future investigation could measure changes in prescribing practices in a larger sample or by means more robust than self-reported behavior and could look for sustained changes in knowledge and confidence past 1 month.

In conclusion, a brief chief resident–led educational intervention increased residents' knowledge and confidence about inpatient management of opioid withdrawal and acute pain in patients on OAT.

Appendices

A. Presentation Materials.pptx

B. Pre- and Postsurvey.docx

All appendices are peer reviewed as integral parts of the Original Publication.

Ayako Wendy Fujita, MD: Fellow, Department of Medicine, Division of Infectious Diseases, Emory University School of Medicine; ORCID: https://orcid.org/0000-0001-8325-1056

Anna LaRosa, MD: Fellow, Department of Medicine, Division of Cardiology, University of Pittsburgh School of Medicine

Andrea Carter, MD, MS: Assistant Professor, Department of Medicine, Division of General Internal Medicine, University of Pittsburgh School of Medicine; Associate Program Director, Internal Medicine Residency Training Program, University of Pittsburgh Medical Center

Acknowledgments
Drs. Fujita and LaRosa contributed equally to this work and are co-primary authors.

Disclosures
None to report.

Funding/Support
The project described was supported by the Clinical Addiction Research and Education (CARE) Program, R25DA013582, as well as the University of Pittsburgh Division of General Internal Medicine.

Ethical Approval
The University of Pittsburgh Institutional Review Board Reported approved this study.

References

1. Rosenthal ES, Karchmer AW, Theisen-Toupal J, Castillo RA, Rowley CF. Suboptimal addiction interventions for patients hospitalized with injection drug use-associated infective endocarditis. *Am J Med*. 2016;129(5):481-485. https://doi.org/10.1016/j.amjmed.2015.09.024
2. Kampman K, Jarvis M. American Society of Addiction Medicine (ASAM) national practice guideline for the use of medications in the treatment of addiction involving opioid use. *J Addict Med*. 2015;9(5):358-367. https://doi.org/10.1097/adm.0000000000000166
3. Kosten TR, O'Connor PG. Management of drug and alcohol withdrawal. *N Engl J Med*. 2003;348(18):1786-1795. https://doi.org/10.1056/nejmra020617
4. Liebschutz JM, Crooks D, Herman D, et al. Buprenorphine treatment for hospitalized, opioid-dependent patients: a randomized clinical trial. *JAMA Intern Med*. 2014;174(8): 1369-1376. https://doi.org/10.1001/jamainternmed.2014.2556
5. Umbricht A, Hoover DR, Tucker MJ, Leslie JM, Chaisson RE, Preston KL. Opioid detoxification with buprenorphine, clonidine, or methadone in hospitalized heroin-dependent patients with HIV infection. *Drug Alcohol Depend*. 2003;69(3):263-272. https://doi.org/10.1016/s0376-8716(02)00325-3
6. Priest KC, Lovejoy TI, Englander H, Shull S, McCarty D. Opioid agonist therapy during hospitalization within the Veterans Health

Copyright © 2021 Fujita et al. This is an open-access publication distributed under the terms of the Creative Commons Attribution license.

Administration: a pragmatic retrospective cohort analysis. *J Gen Intern Med*. 2020;35(8):2365-2374. https://doi.org/10.1007/s11606-020-05815-0

7. Alford DP, Bridden C, Jackson AH, et al. Promoting substance use education among generalist physicians: an evaluation of the Chief Resident Immersion Training (CRIT) program. *J Gen Intern Med*. 2009;24(1):40-47. https://doi.org/10.1007/s11606-008-0819-2
8. Tesema L, Marshall J, Hathaway R, et al. Training in office-based opioid treatment with buprenorphine in US residency programs: a national survey of residency program directors. *Subst Abus*. 2018;39(4):434-440. https://doi.org/10.1080/08897077.2018.1449047
9. Alford DP, Carney BL, Brett B, Parish SJ, Jackson AH. Improving residents' safe opioid prescribing for chronic pain using an objective structured clinical examination. *J Grad Med Educ*. 2016;8(3):390-397. https://doi.org/10.4300/jgme-d-15-00273.1
10. Kunins HV, Sohler NL, Giovanniello A, Thompson D, Cunningham CO. A buprenorphine education and training program for primary care residents: implementation and evaluation. *Subst Abus*. 2013;34(3):242-247. https://doi.org/10.1080/08897077.2012.752777
11. Pasquale MK, Sheer RL, Mardekian J, et al. Educational intervention for physicians to address the risk of opioid abuse. *J Opioid Manag*. 2017;13(5):303-313. https://doi.org/10.5055/jom.2017.0399
12. Jennings L, Warner T, Bacro-Duverger B. Identification and treatment of opioid withdrawal and opioid use disorder in the emergency department. *MedEdPORTAL*. 2020;16:10899. https://doi.org/10.15766/mep_2374-8265.10899
13. Klimas J, Ahamad K, Fairgrieve C, et al. Impact of a brief addiction medicine training experience on knowledge self-assessment among medical learners. *Subst Abus*. 2017;38(2):141-144. https://doi.org/10.1080/08897077.2017.1296055

Received: August 12, 2020
Accepted: January 10, 2021
Published: March 10, 2021

Copyright © 2021 Fujita et al. This is an open-access publication distributed under the terms of the Creative Commons Attribution license.
